# Supplementary material for: Highly biased agonism for GPCR ligands via nanobody tethering
Source: Nat Commun. 2024 Jun 1;15:4687. doi: 10.1038/s41467-024-49068-5 (PMC11144202; doi:10.1038/s41467-024-49068-5)
Supplement: Supplementary file 1 — Supplementary Information [file 41467_2024_49068_MOESM1_ESM.pdf]

Supplementary Information for

Highly biased agonism for GPCR ligands via nanobody tethering

Shivani Sachdev[1], Brendan A. Creemer[1], Thomas J. Gardella[2], Ross W. Cheloha*[1]

[1]Laboratory of Bioorganic Chemistry; National Institutes of Diabetes, Digestive, and Kidney Diseases; National Institutes of Health, USA. [2]Endocrine Unit, Massachusetts General Hospital and Harvard Medical School, Boston, Massachusetts, USA.

*Email: ross.cheloha@nih.gov

Supplementary Tables:

Peptide	$[M+H]_{calc}$	$[M+H]_{obs}$
$PTH_{1\text{-}34}$-Cys	4218	4220
$PTH_{1\text{-}11}$-Cys	1412	1412
$PTH_{1\text{-}11}$-Gly	1366	1366
$PTH_{1\text{-}11}$-DBCO	1839	1839
$PTH_{1\text{-}11}$-PEG_4-DBCO	2086	2086
$PTH_{1\text{-}21}$-Cys	2667	2667
$PTH_{1\text{-}11}$-6E	3047	3046
G_3-exon2	2175	2175
$PTH_{1\text{-}11}$-$(Ahx)_2$-6E	3160	3160
$PTH_{1\text{-}11}$-Cys(TMR)-Ahx-azide	2231	2231
$PTH_{1\text{-}34}$-TMR	4770	4771
$GLP1_{mut}$	3340	3340
$GLP1_{mut}$-DBCO	4016	4016

Supplementary Table 1: Mass spectroscopy characterization of the peptides used in this study. Peptides were analyzed by LC/MS as described in methods. Calculated masses ($[M+H]_{calc}$) refers to the monoisotopic mass of a singly protonated species. The masses recorded using mass spectrometry are labeled as $[M+H]_{obs}$. Mass spectra are provided in Supplementary Data File 1.

Conjugate	$[M+H]_{calc}$	$[M+H]_{obs}$
Nb_{6E}	-	13475
Nb_{6E}-biotin-azide	13221	13216
PTH_{1-11}-Nb_{6E}	15302	15324
Nb_{PTHR1}	-	14467
Nb_{PTHR1}-DBCO	14230	14230
Nb_{PTHR1}-biotin-azide	14210	14207
PTH_{1-11}-Nb_{PTHR1}	16047	16050
Nb_{PTHR1}-TMR	14355	14356
PTH_{1-11}-Nb_{PTHR1}-TMR	16461	16466
$Nb_{PTHR1X2}$	-	14703
$Nb_{PTHR1X2}$-TMR	14593	14607
$Nb_{PTHR1X2}$-biotin-azide	14443	14443
PTH_{1-11}-$Nb_{PTHR1x2}$	16283	16284
Nb_{GFP}	-	14232
Nb_{GFP}-biotin-azide	13978	13973
Nb_{MHC-I}	-	14440
Nb_{MHC-I}-biotin-azide	14189	14184
Nb_{neg} (BC-2[1])	-	14993
Nb_{neg}-biotin-azide	14846	14845
PTH_{1-11}-Nb_{neg}	16683	16684
PTH_{1-11}-PEG_4-Nb_{PTHR1}	16293	16298
Nb_{GLP1R}	-	14844
Nb_{GLP1R}-biotin-azide	14586	14586
$GLP1_{mut}$-Nb_{GLP1R}	18600	18601
$GLP1_{mut}$-Nb_{6E}	17236	17253
$GLP1_{mut}$-Nb_{neg}	18589	18586

Supplementary Table 2: Confirmation of Nb-peptide conjugate identity using mass spectrometry. Nb-peptide conjugates were analyzed by LC/MS as described in Methods. MW_{calc} refers to the calculated average molecular weight and MW_{obs} refers to the molecular weight recorded by mass spectrometry following analysis using MaxENT for deconvolution. Nb_{Neg} corresponds to a nanobody that binds to the BC2 epitope, used here as a negative control[1]. Unlike the other Nbs used in this study, Nb_{neg} was site-specifically modified with G_3-biotin-Ahx-azide instead of G3-biotin-azide during the sortagging reaction. Dashes correspond to unmodified nanobody molecular weights, for which only experimentally observed masses were used for further calculations.

Independent experiment (N)	cAMP	Washout AUC	Gαs	β-arrestin 2 (plasma membrane)	β-arrestin 2 (endosome)	Intracellular calcium changes
**PTHR1-6E**						
$PTH_{1\text{-}34}$	6	6	4	4	3	3
$PTH_{1\text{-}11}$	6	6	4	4	3	3
$PTH_{1\text{-}11}$-Nb_{6E}	6	6	4	4	3	3
$PTH_{1\text{-}11}$-Nb_{neg}	6	6	4	4	3	3
**PTHR1-Nb_{6E}**						
$PTH_{1\text{-}34}$	4	4	3	6	3	3
$PTH_{1\text{-}11}$	4	4	3	6	3	3
$PTH_{1\text{-}11}$-Ahx-6E	4	4	3	6	3	3
**PTHR1**						
$PTH_{1\text{-}34}$	5	5	3	6	3	3
$PTH_{1\text{-}11}$	5	5	3	6	3	3
$PTH_{1\text{-}11}$-Nb_{PTHR1}	5	5	3	6	3	3
$PTH_{1\text{-}11}$-$Nb_{PTHR1\mathbf{X2}}$	3	3	0	3	0	0

Supplementary Table 3: Quantity of independent replicate experiments performed for both ligands and conjugates for data shown in Table 1 in the main text.

Supplementary Figures:

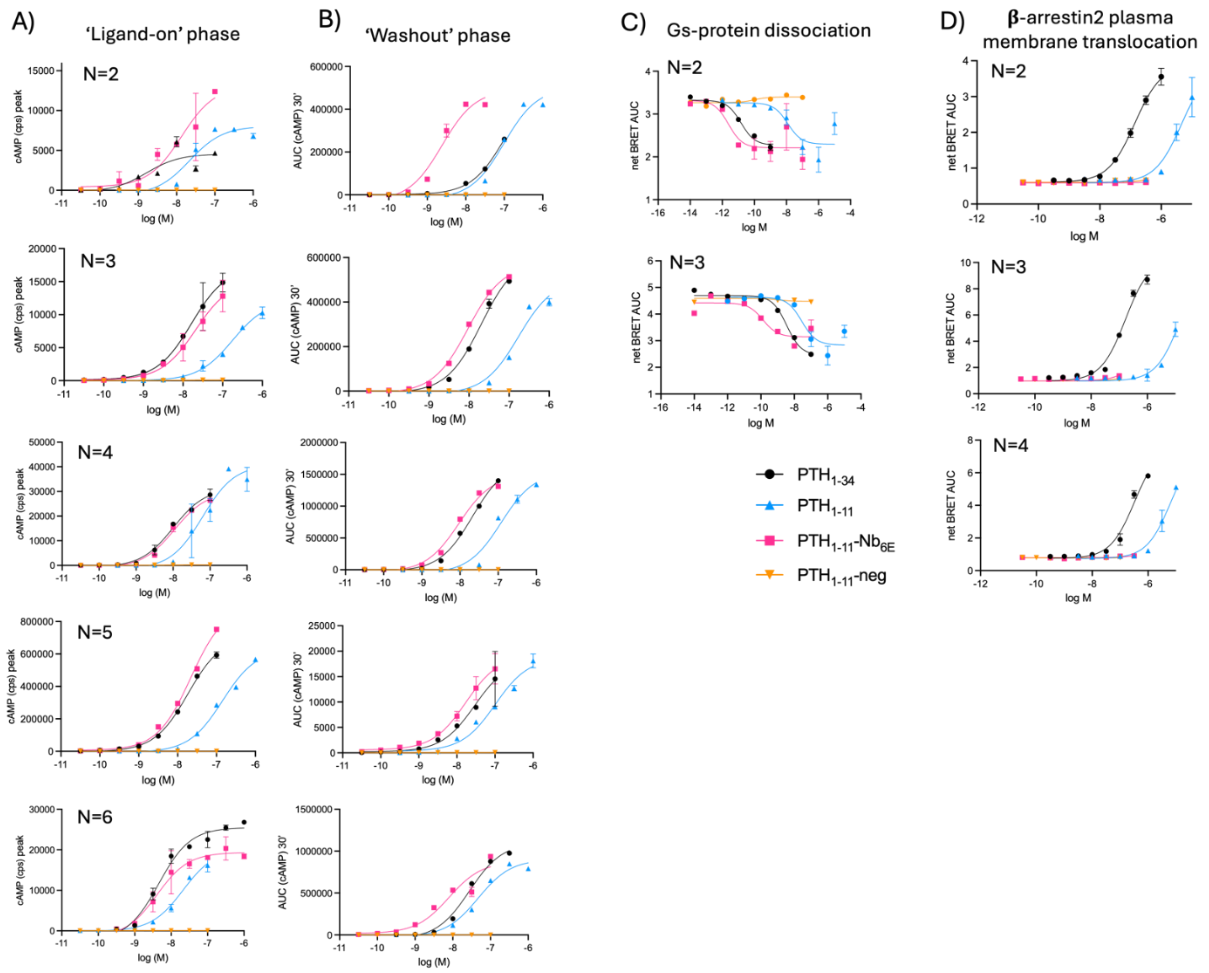

Supplementary Figure 1: Independent experiments for ligand activity on engineered PTHR1-6E receptor. Dose response curves for ligand conjugates on A) cAMP responses, B) washout responses generated from quantifying area under curve, C) Gαs-protein dissociation from plasma membrane, and D) β-arrestin2 recruitment to the plasma membrane were measured in HEK cells transfected and selected to stably express PTHR1-6E. Curves were generated by fitting to a three-parameter logistic equation (separate from that shown in main Figure 2 and 3).

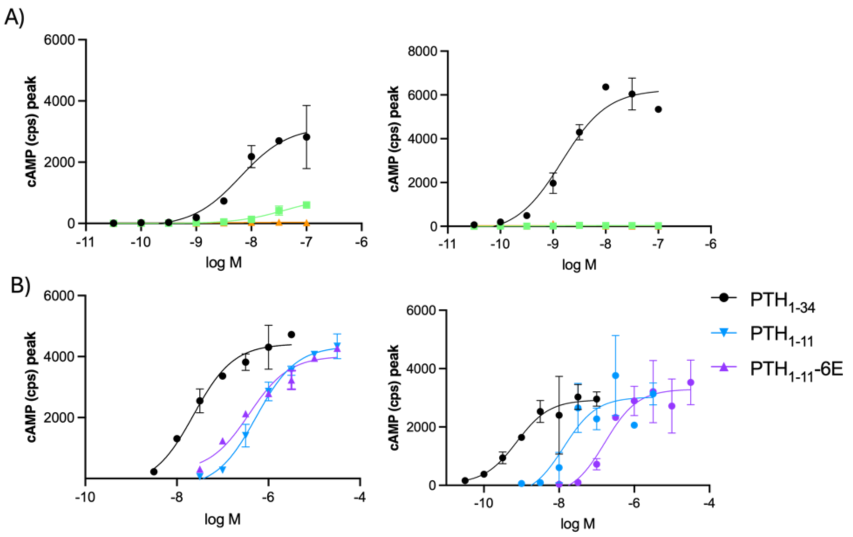

C)

EC50 (±SD) Ligands, nM	cAMP
PTH_{1-34}	5 (±3)
PTH_{1-11}	23 (± 9)
PTH_{1-11}-6E	214 (±107)

Supplementary Figure 2: Evaluation of ligand bioactivity in the absence of Nb or peptide tethering. Indicated peptides and ligands were evaluated on the HEK-PTHR1 cell line for the induction of cAMP responses. A) Representative concentration-response curves for Nb-PTH_{1-11} conjugates comprised of Nbs that do not bind to WT PTHR1. B) Representative concentration-response curves for peptides in cells expressing WT PTHR1. Each graph corresponds to an independent experiment with data points representing mean ± SD from technical duplicates. C) Tabulation of pharmacological parameters for indicated peptides. EC_{50} values correspond to mean (±SD) measurements from 3 independent biological replicates.

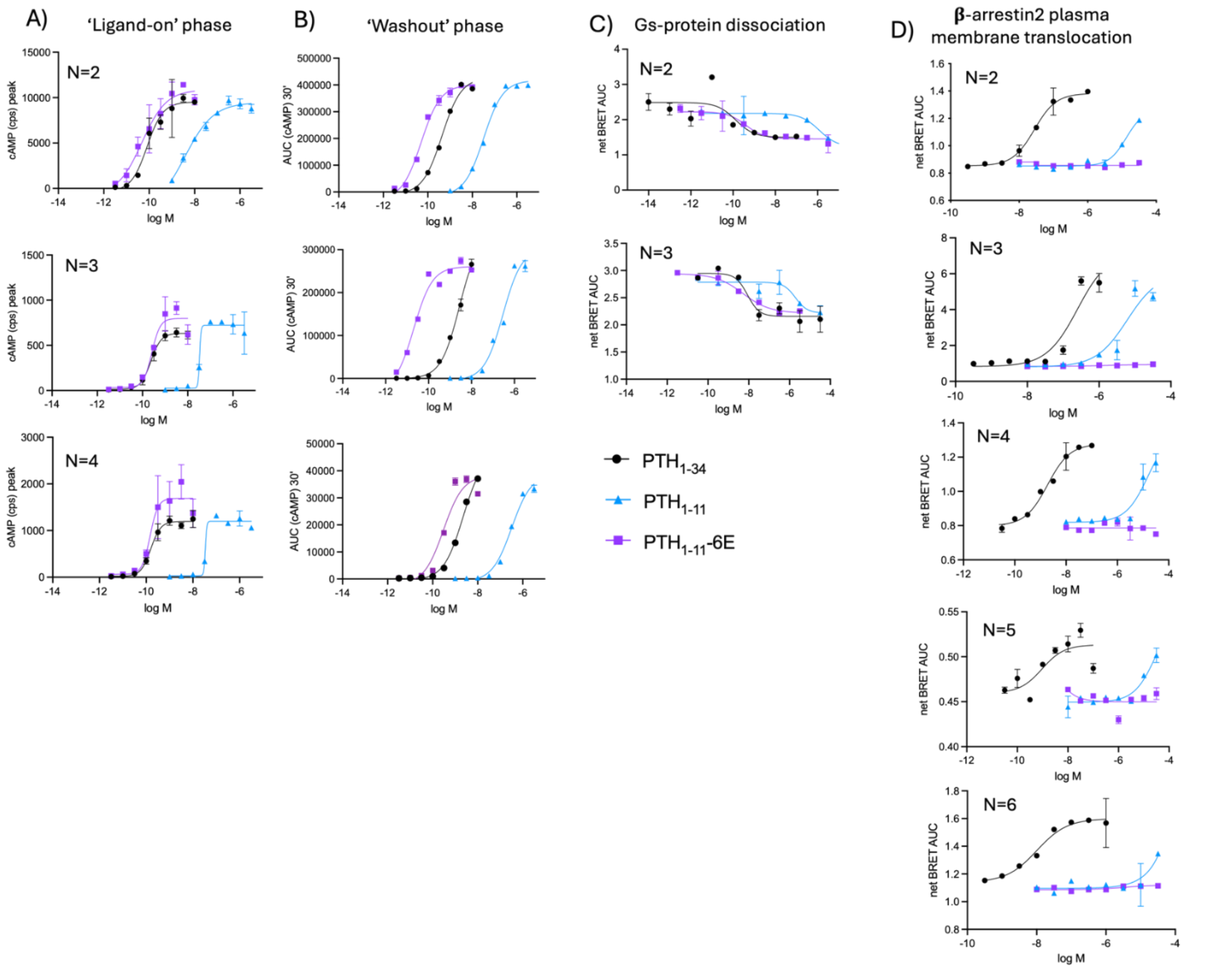

Supplementary Figure 3: Independent experiments for ligand activity on engineered PTHR1-Nb$_{6E}$ receptor. Dose response curves for ligand conjugates on A) cAMP responses, B) washout responses generated from quantifying area under curve, C) Gαs-protein dissociation from plasma membrane, and D) β-arrestin2 recruitment to the plasma membrane were measured in HEK cells transfected and selected to stably express PTHR1-Nb$_{6E}$. Curves were generated by fitting to a three-parameter logistic equation (separate from that shown in main Figure 2 and 3).

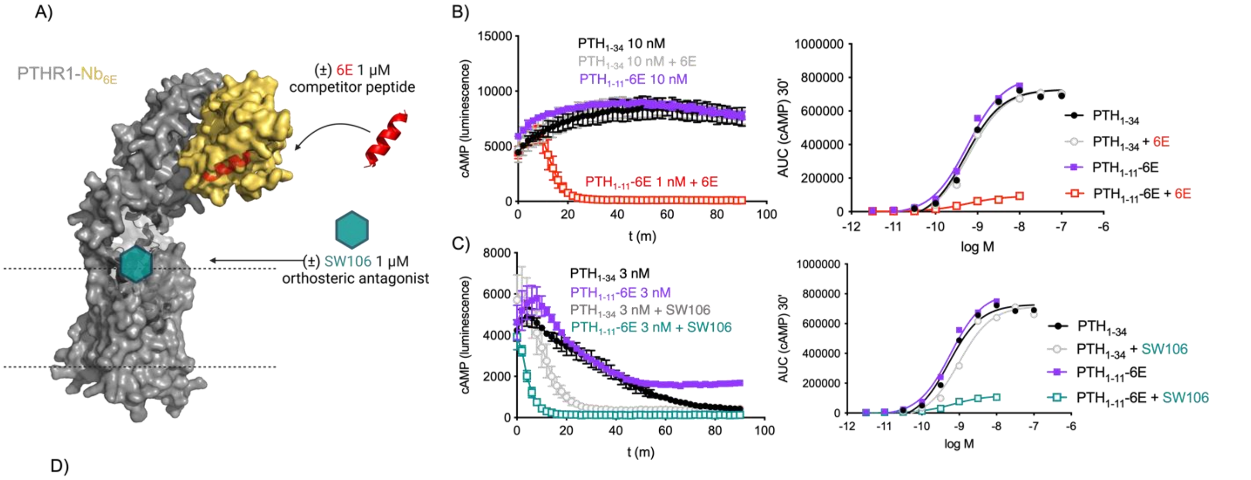

D)

Ligands	cAMP, Washout AUC					
	Control		+6E		+SW106	
	EC_{50} (±SEM), nM	% $PTH_{1\text{-}34}$ activation E_{max} (±SEM)	EC_{50} (±SEM), nM	% $PTH_{1\text{-}34}$ activation E_{max} (±SEM)	EC_{50} (±SEM), nM	% $PTH_{1\text{-}34}$ activation E_{max} (±SEM)
$PTH_{1\text{-}34}$	1.2 (0.7)	100	0.7 (0.3)	100	2.2 (3.7)	100
$PTH_{1\text{-}11}$-6E	0.18 (0.12)	110 (15)	-	16 (6)	-	13 (7)

Supplementary Figure 4: Impact of antagonists on washout responses of ligand conjugates A) Schematic of the washout competition assay for PTHR1-Nb_{6E} in the continued presence of synthetic 6E peptide or orthosteric PTHR1 antagonist, SW106. B) Time course for cAMP production in HEK293 cells stably expressing PTHR1-Nb_{6E} after washout of ligands in the presence or absence of competitor 6E peptide. Data sets in the right correspond to values generated from quantifying the area under the curve for kinetic washout responses. C) Analogous data for cAMP washout responses in the presence or absence of antagonist, SW106. Data points correspond to mean and associated SD from technical replicates. D) Compiled tabulation of agonist potency and Emax parameters derived from 3 independent experiments. For compounds where a plateau was not reached, Emax corresponds to the response observed at the highest dose. Data correspond to presented as mean (±SEM). A dash indicates activity was too weak to calculate an EC_{50} value.

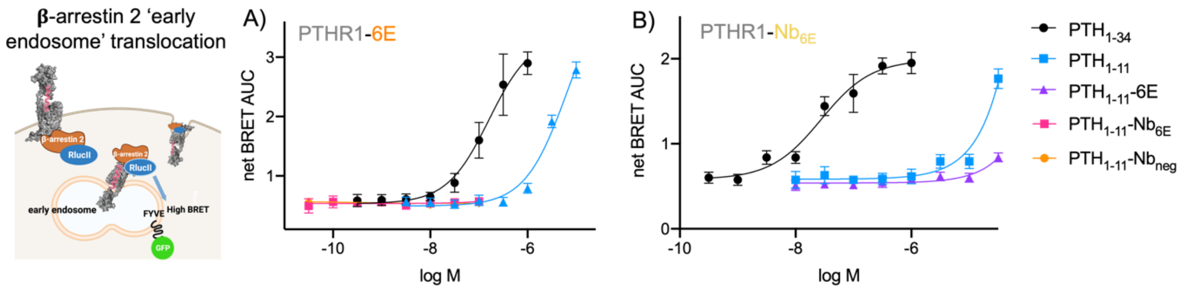

Supplementary Figure 5: Characterization of endosomal β-arrestin 2 recruitment by ligand conjugates. A) Stimulation of β-arrestin 2 translocation to endosomes in HEK cells expressing PTHR1-6E. An increase in BRET ratio indicates ligand-induced β-arrestin 2 translocation to endosomes. Data are presented as AUC generated from BRET kinetic measurements. B) Analogous data for cells expressing PTHR1-Nb_{6E}. Data points correspond to mean and SD from technical replicates in a representative experiment, fit to a three-parameter logistic sigmoidal model. Tabulation of agonist potency parameters are shown in main Table 1, derived from 3-5 independent experiments. This figure was created in part with BioRender.com released under a Creative Commons Attribution-NonCommercial-NoDerivs 4.0 International license: https://creativecommons.org/licenses/by-nc-nd/4.0/deed.en

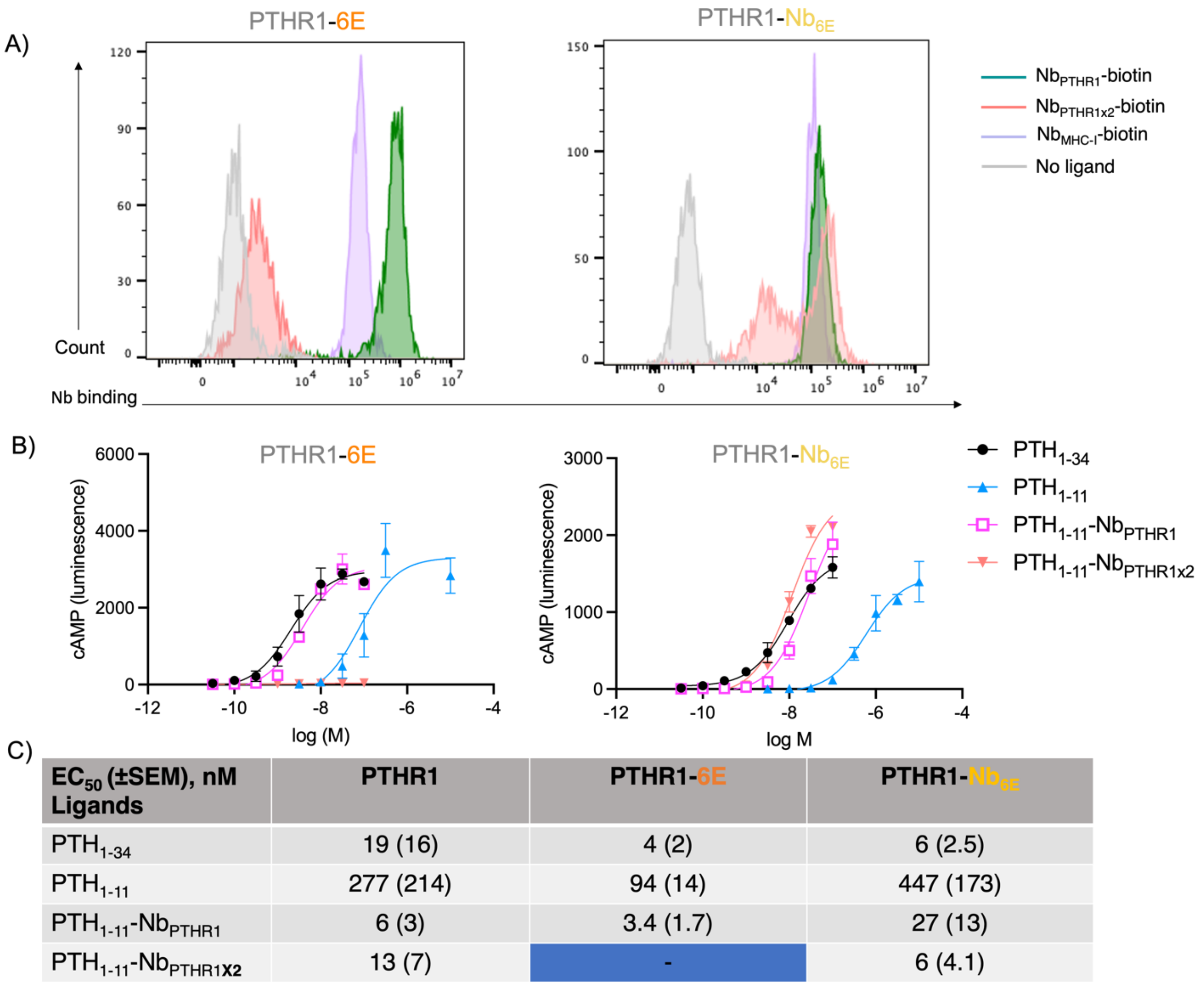

C)

EC_{50} (±SEM), nM Ligands	PTHR1	PTHR1-6E	PTHR1-Nb_{6E}
$PTH_{1\text{-}34}$	19 (16)	4 (2)	6 (2.5)
$PTH_{1\text{-}11}$	277 (214)	94 (14)	447 (173)
$PTH_{1\text{-}11}$-Nb_{PTHR1}	6 (3)	3.4 (1.7)	27 (13)
$PTH_{1\text{-}11}$-$Nb_{PTHR1X2}$	13 (7)	-	6 (4.1)

Supplementary Figure 6: Assessment of Nb_{PTHR1} and $Nb_{PTHR1X2}$ acting on engineered receptors. A) Representative histograms for flow cytometry analysis of Nb_{PTHR1} and $Nb_{PTHR1\text{-}X2}$ binding to PTHR1-6E and PTHR1-Nb_{6E} receptors. Nbs (500 nM) labeled with biotin were incubated with cells expressing PTHR1-6E or PTHR1-Nb_{6E}, followed by washing, detection with streptavidin-APC, and assessment of cellular fluorescence. No ligand refers to cells not exposed to biotin-labeled Nbs. $Nb_{MHC\text{-}I}$ is included for comparison to the staining intensity observed with a highly expressed cell surface protein. B) Dose-response curves for maximal cAMP responses generated upon addition of ligands to cells expressing indicated receptors. Curves were generated by fitting to a 3-parameter logistic equation. C) Compiled tabulation of agonist potency parameters for ligand-conjugates. EC_{50} values correspond to mean (±SEM) measurements from 3 or more assays run in duplicates. A dash indicates activity was too weak to calculate an EC_{50} value. Note that a subset of this data (i.e. EC_{50} for $PTH_{1\text{-}34}$ and $PTH_{1\text{-}11}$) are duplicated in the main text Table 1.

A)

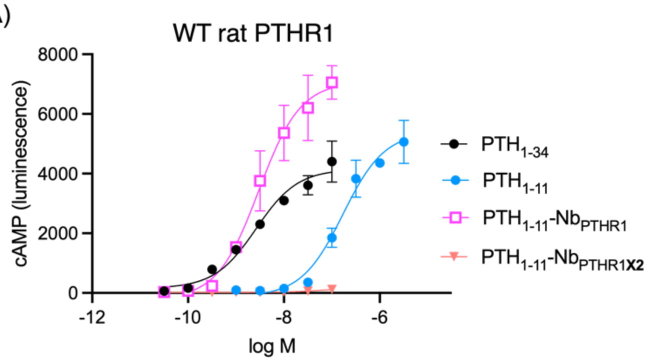

B)

Ligands	Rat PTHR1, cAMP EC_{50} (±SEM), nM
PTH_{1-34}	6.6 (3)
PTH_{1-11}	117 (40)
PTH_{1-11}-Nb_{PTHR1}	1.9 (0.7)
PTH_{1-11}-$Nb_{PTHR1X2}$	-

Supplementary Figure 7: Dose response data for Nb-ligand conjugates for cAMP production was assessed on cells expressing WT rat PTHR1. A) Curves were generated using a three-parameter logistic sigmoidal model from a single representative experiment. B) Compiled tabulation of agonist potency parameters for ligand-conjugates at WT rat PTHR1. EC_{50} values correspond to mean (±SEM) measurements from 3 independent biological replicates. A dash indicates activity was too weak to calculate an EC_{50} value.

A)

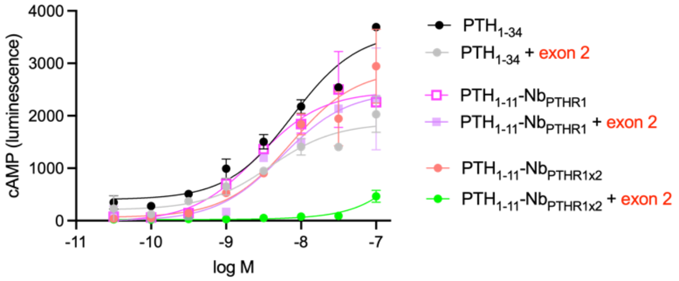

B)

PTHR1	EC_{50}, nM	SEM
without exon 2		
PTH_{1-34}	8	0.5
PTH_{1-11}-Nb_{PTHR1}	4.3	2.2
PTH_{1-11}-$Nb_{PTHR1X2}$	6.1	1.2
with exon 2		
PTH_{1-34}	5.1	2.3
PTH_{1-11}-Nb_{PTHR1}	4	1
PTH_{1-11}-$Nb_{PTHR1X2}$	>100	-

Supplementary Figure 8: Blockade of $Nb_{PTHR1-X2}$ binding through exogenous peptide derived from PTHR1 exon 2. A) Dose response data for Nb-ligand conjugates for cAMP production was assessed on WT PTHR1 treated with synthetic exon 2 peptide (GGGWTSASTSGKPRKDKASGKL, 1.7 μM). This peptide encompasses the sequence of the portion of PTHR1 replaced by epitope tag in PTHR1-6E. Curves were generated using a three-parameter logistic sigmoidal model from a single representative experiment with or without exon 2 peptide. B) Compiled tabulation of agonist potency parameters for ligand-conjugates at PTHR1. EC_{50} values correspond to mean (±SEM) measurements from 3 biological replicates. A dash indicates activity was too weak to calculate an EC_{50} value.

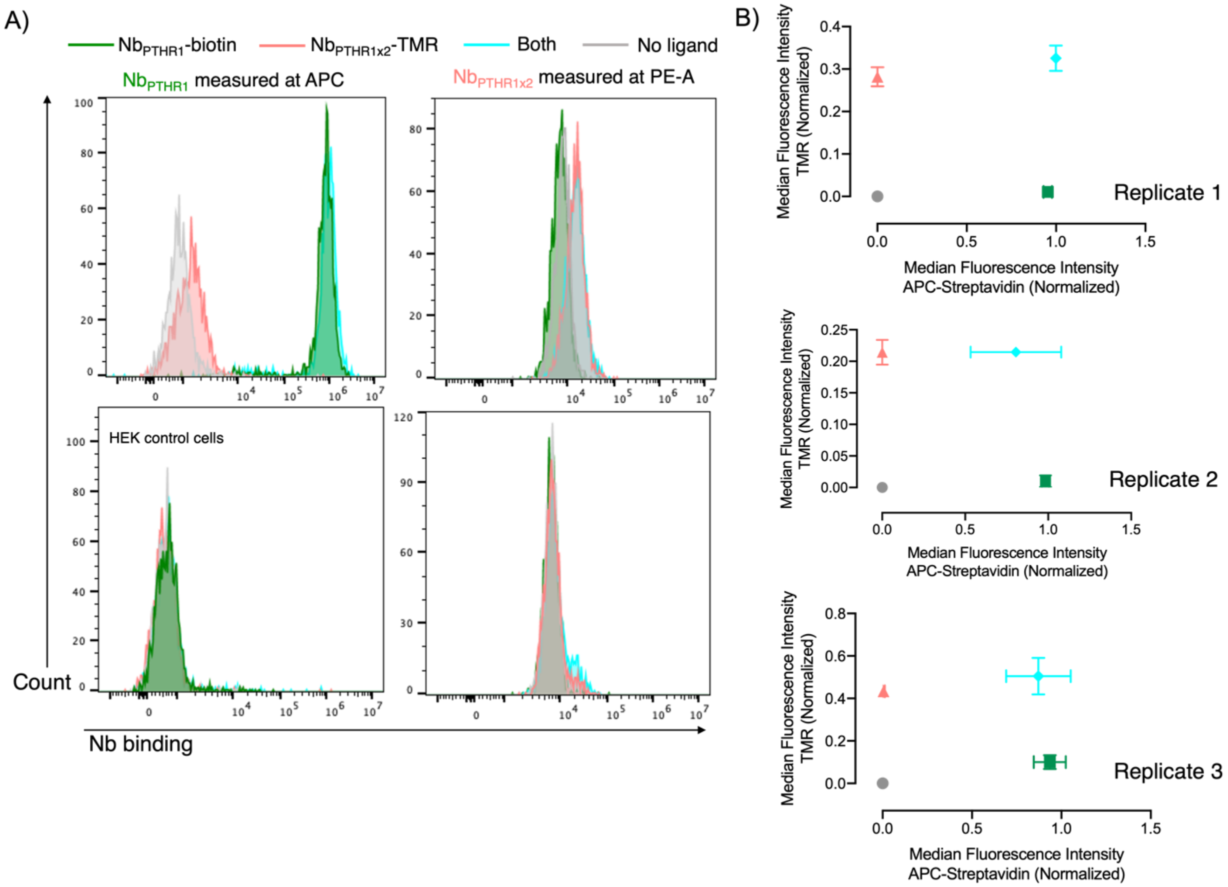

Supplementary Figure 9: Assessment of Nb$_{PTHR1}$ and Nb$_{PTHR1-X2}$ binding. A) Representative histograms for flow cytometry analysis of Nb$_{PTHR1}$ and Nb$_{PTHR1-X2}$ with distinct labels binding to PTHR1. Nb$_{PTHR1}$ labeled with biotin (300 nM) and Nb$_{PTHR1-X2}$ labeled with TMR (300 nM) were incubated with cells expressing PTHR1, followed by washing, detection with streptavidin-APC, and measurement. “No ligand” refers to cells not exposed to labeled Nbs. “Both” refers to simultaneous treatment of cells with two distinctly labeled Nbs added together. The histogram in the bottom row of panels represents the same labeling experiment run on untransfected cells that do not express PTHR1. B) Quantified data normalized to the maximum signal observed for an index ligand presented as median fluorescence intensity values. Each plot corresponds to an independent experiment. Data points correspond to mean ± SD for technical replicates in each experiment.

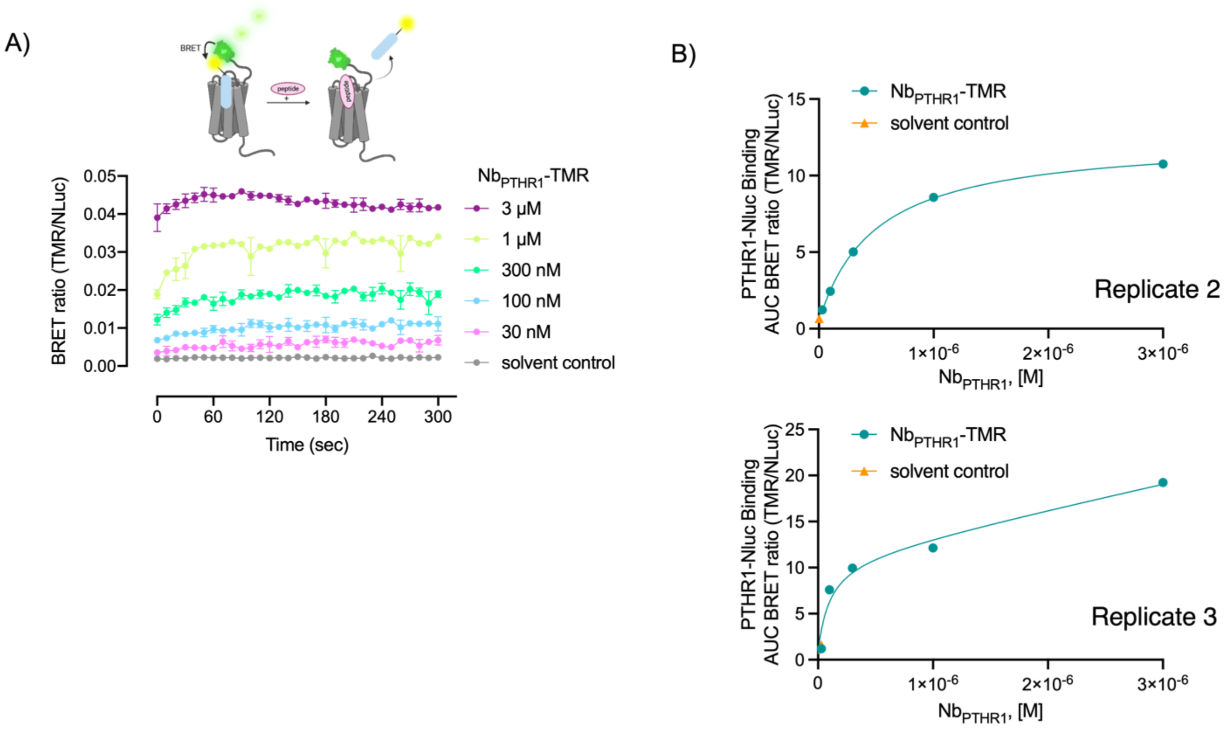

Supplementary Figure 10: Evaluating Nb$_{PTHR1}$ binding to PTHR1 using BRET. A) Schematic and data corresponding to BRET kinetic measurements for the binding (association) of increasing concentrations of Nb$_{PTHR1}$-TMR to HEK cells expressing nLuc-PTHR1. Data are shown as mean ± SD from technical replicates in a single representative experiment. Note that by the time measurements begin the BRET signal is already at a plateau level, precluding calculation of association and dissociation kinetics. B) Concentration-response curves for biological replicates of BRET measurements of Nb$_{PTHR1}$-TMR binding to nLuc-PTHR1. Data points correspond to quantification of the area under the curve for kinetic association curves. Data are fitted to a 3-parameter sigmoidal dose response model. The third technical replicate is found in the main text (Figure 4B). Panel A was created in part with BioRender.com released under a Creative Commons Attribution-NonCommercial-NoDerivs 4.0 International license: https://creativecommons.org/licenses/by-nc-nd/4.0/deed.en

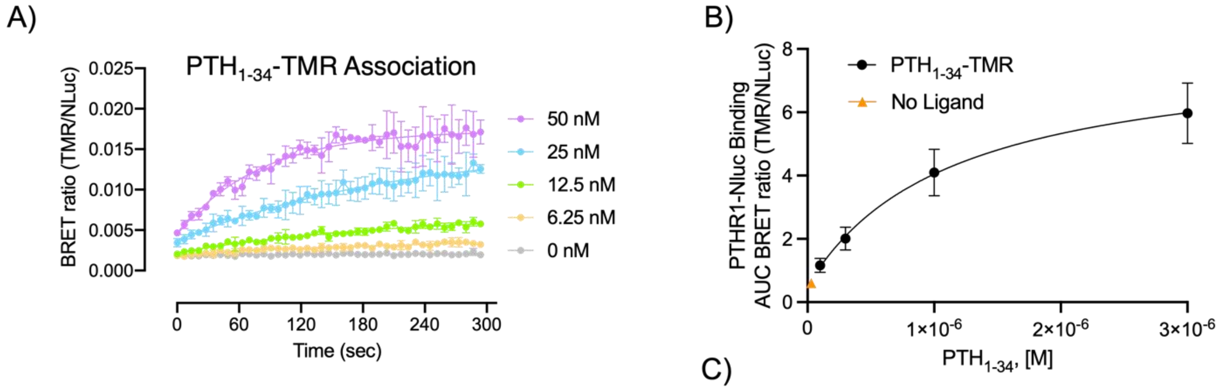

C)

PTH_{1-34}	
k_{on} ($\times 10^5$ $s^{-1}M^{-1}$)	3.6 ± 0.3
k_{off} (s^{-1})	0.003 ± 0.0002
K_d (nM)	14 ± 1

Supplementary Figure 11: Evaluation of PTH_{1-34} binding to PTHR1 using BRET. A) Representative BRET kinetic measurements of the binding (association) of increasing concentrations of PTH_{1-34}-TMR (0-50 nM) added at time zero to HEK cells expressing nLuc-PTHR1. Data are shown as mean ± SD from technical replicates in a single representative experiment. B) Representative concentration-response curve for BRET measurements of PTH_{1-34}-TMR binding to nLuc-PTHR1. Data set generated from quantifying the area under the curve for kinetic association curve. Data are fitted to a 3-parameter sigmoidal dose response model. C) Tabulation of kinetic parameters and dissociation constants of PTH_{1-34} binding to nLuc-PTHR1 fitted to a one-phase association model (see Methods). Kinetic parameters correspond to means ± SEM from 3 independent experiments.

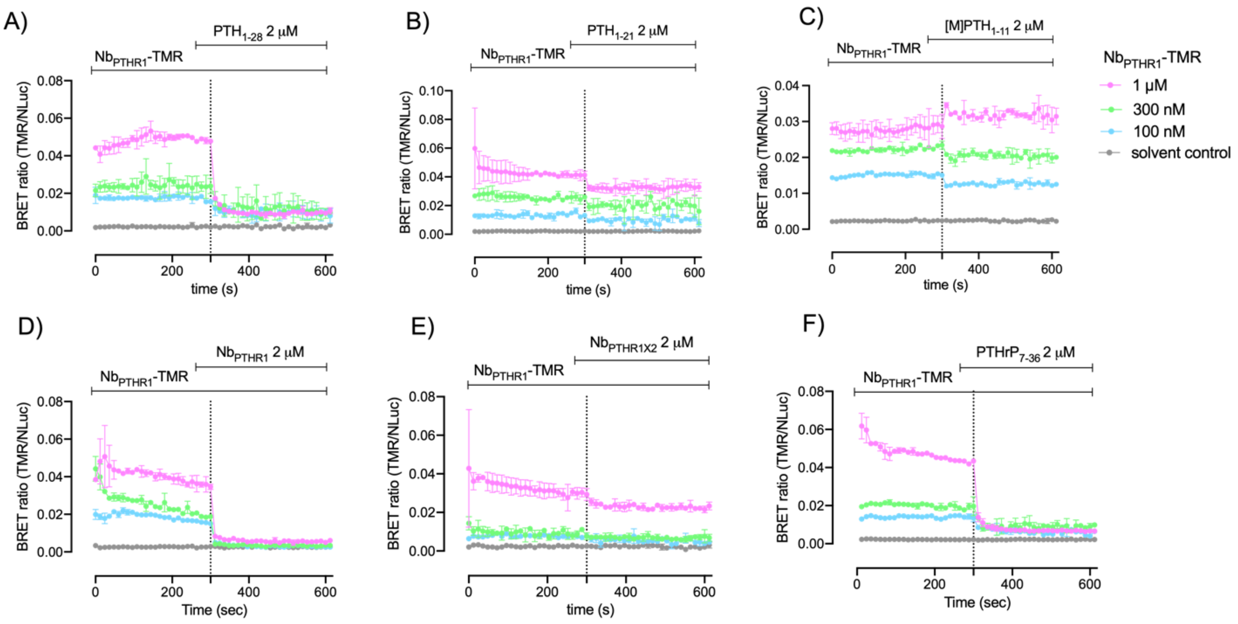

Supplementary Figure 12: Kinetic traces of BRET binding experiments for the competition of Nb_{PTHR1}-TMR with unlabeled competitors. Representative BRET kinetic traces of Nb binding observed upon application of varying concentrations of Nb_{PTHR1}-TMR followed by addition of unlabeled ligand (A) PTH_{1-28}, (B) PTH_{1-21}, (C) PTH_{1-11}, (D) Nb_{PTHR1}, (E) $Nb_{PTHR1\text{-}\mathbf{X2}}$, and (F) $PTHrP_{7-36}$ at a concentration of 2 μM. Quantified summaries for the these experiments is shown in Figure 4C. Data points correspond to mean ± SD from technical replicates in a single representative experiment.

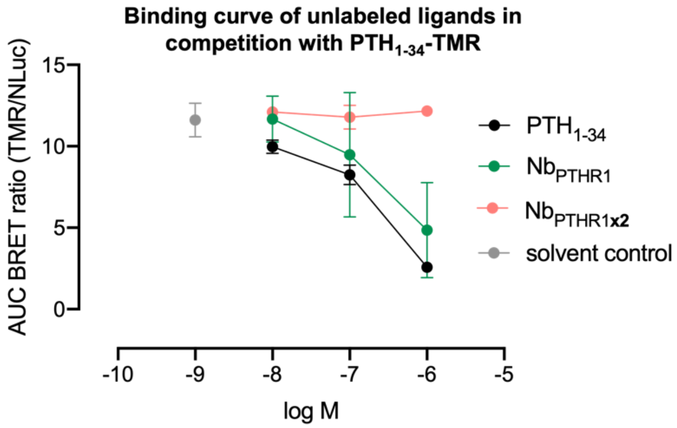

Supplementary Figure 13: Competition BRET binding assays with PTH_{1-34}-TMR. Concentration-response competition binding assays were performed using varying concentration of unlabeled Nbs (or peptide) added simultaneously with PTH_{1-34}-TMR (300 nM). Data points correspond to mean (BRET AUC) ± SEM from 3 independent experiments.

PTHR1-ECD: Nb_{PTHR1}

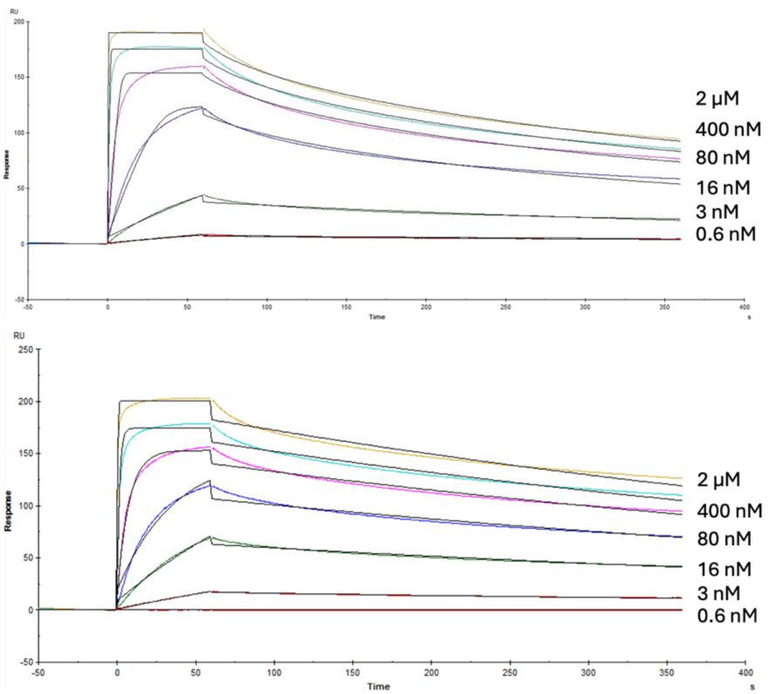

Kinetic parameters:
$k_a = 1.2 *10^7 M^{-1}s^{-1}$
$k_d = 6.5 *10^{-3} s^{-1}$
$K_D = 0.5$ nM

Kinetic parameters:
$k_a = 1.6 *10^6 M^{-1}s^{-1}$
$k_d = 1.5 *10^{-3} s^{-1}$
$K_D = 0.9$ nM

Mean (±SD), nM	
K_D	0.7 (±0.3)

Supplementary Figure 14: Characterization of Nb_{PTHR1} binding to PTHR1-ECD by SPR assays. Sensorgrams showing PTHR1-ECD association and dissociation to immobilized Nb_{PTHR1}. Nb_{PTHR1} was biotinylated at its C-terminus through sortase-mediated ligation and immobilized onto streptavidin-coated sensor chips. k_a and k_d are the association and dissociation rate constants from individual experiments; K_D is the equilibrium dissociation constant. The tabulated K_D corresponds to mean (±SD) derived from the two experiments shown in this figure. Experimental data are shown in color and compared with modeled curves shown in black.

PTHR1-ECD:

GDDVMTKEEQIFLLHRAQAQCEKRLKEVLQRPASIMESDKGWTSASTSGKPRKDKAS
GKLYPESEEDKEAPTGSRYRGRPCLPEWDHILCWPLGAPGEVVAVPCPDYIYDFNHK
GHAYRRCDRNGSWELVPGHNRTWANYSECVKFLTNETREREVFDRL*

GLP1R-ECD:

RPQGATVSLWETVQKWREYRRQCQRSLTEDPPPATDLFCNRTFDEYACWPDGEPGS
FVNVSCPWYLPWASSVPQGHVYRFCTAEGLWLQKDNSSLPWRDLSECEESKRGERS
SPEEHHHHHH

Supplementary Figure 15: Sequence for PTHR1 and GLP1R extracellular domain construct used in this study. The PTHR1 extracellular domain construct was produced and purified as described in Methods. GLP1R-ECD construct was obtained from a commercial vendor (GenScript) using recombinant expression in Chinese Hamster Ovary (CHO) cells.

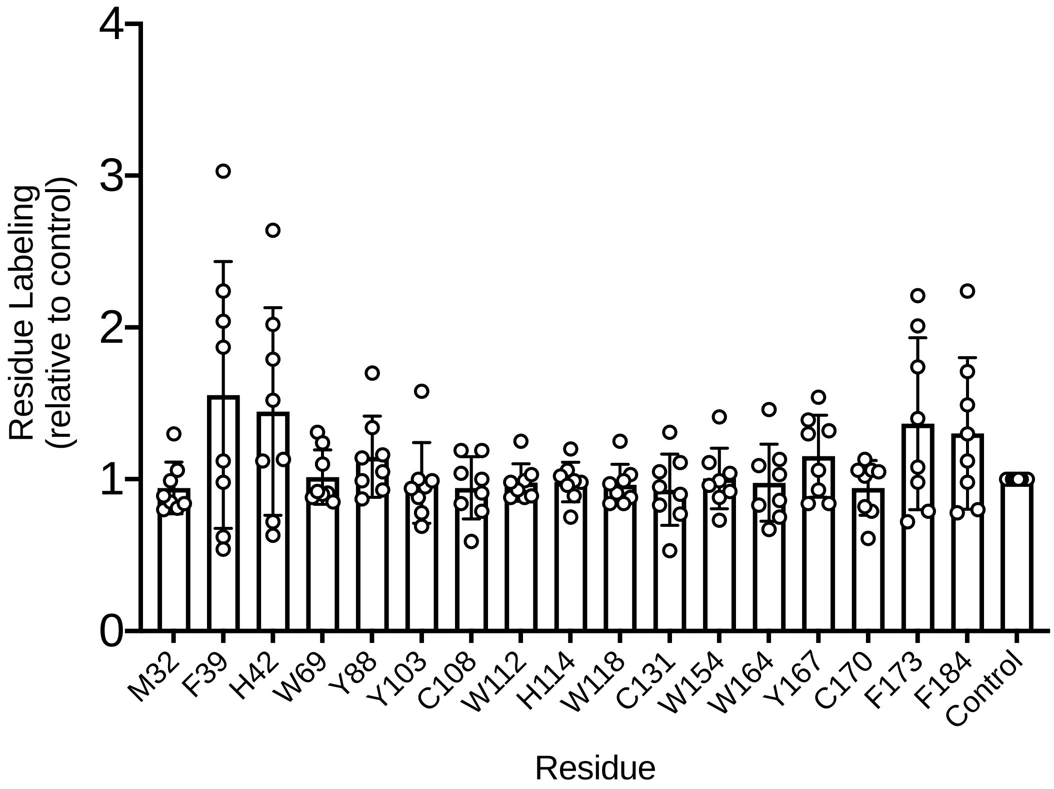

Supplementary Figure 16: Analysis of Nb_{PTHR1}-PTHR1 ECD interactions using hydroxy radical-based footprinting analysis. Data points correspond to 8 independent replicates ± SD. Labeling is normalized to a control performed in the presence of a non-binding Nb (Nb_{6E}). See Figure 4E for residues showing a reduction in binding upon addition of Nb_{PTHR1}.

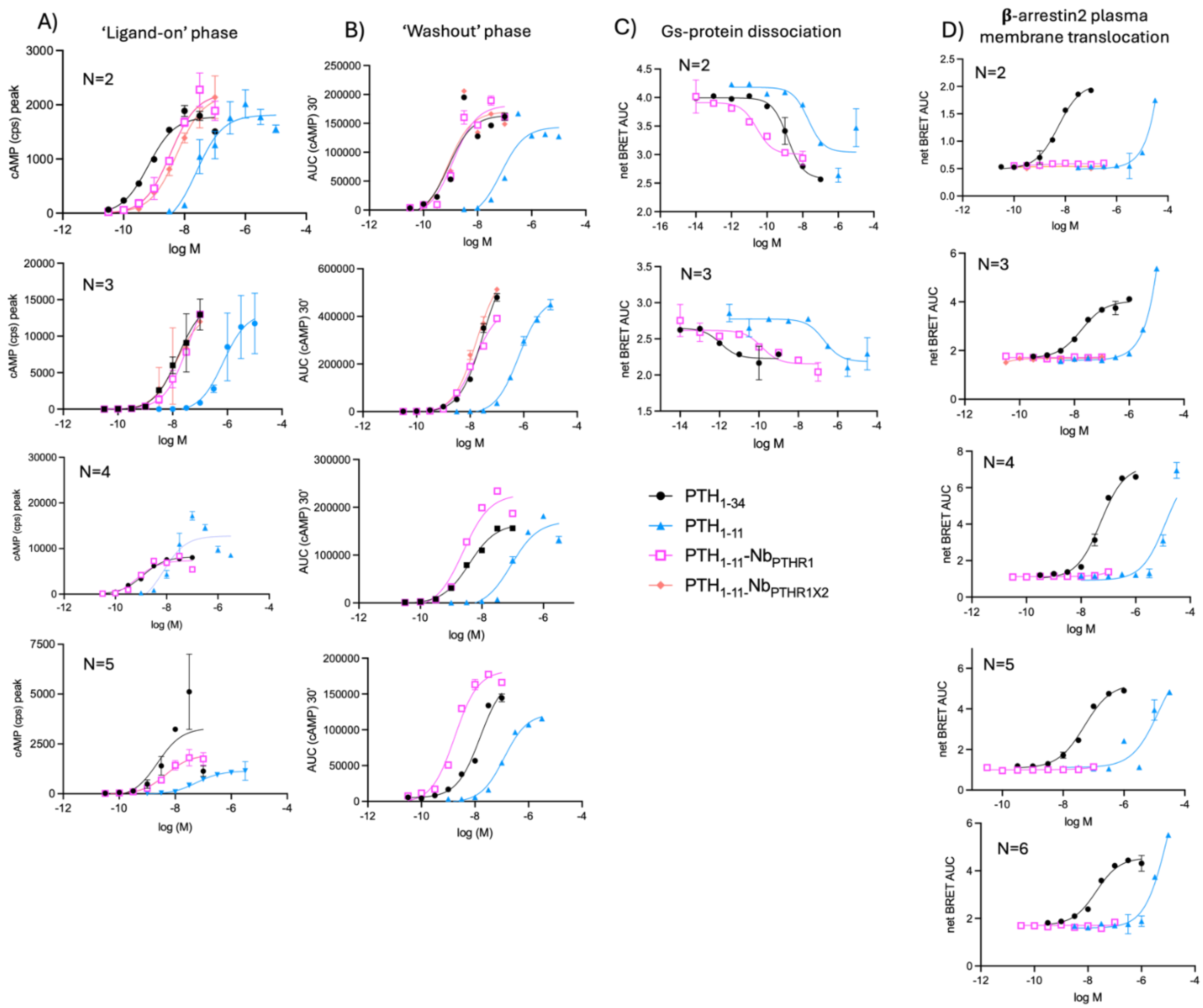

Supplementary Figure 17: Independent experiments for ligand activity on WT PTHR1 receptor. Dose response curves for ligand conjugates on A) cAMP responses, B) washout responses generated from quantifying area under curve, C) Gαs-protein dissociation from plasma membrane, and D) β-arrestin2 recruitment to the plasma membrane were measured in HEK cells transfected and selected to stably express WT PTHR1. Curves were generated by fitting to a three-parameter logistic equation (separate from that shown in main Figure 5).

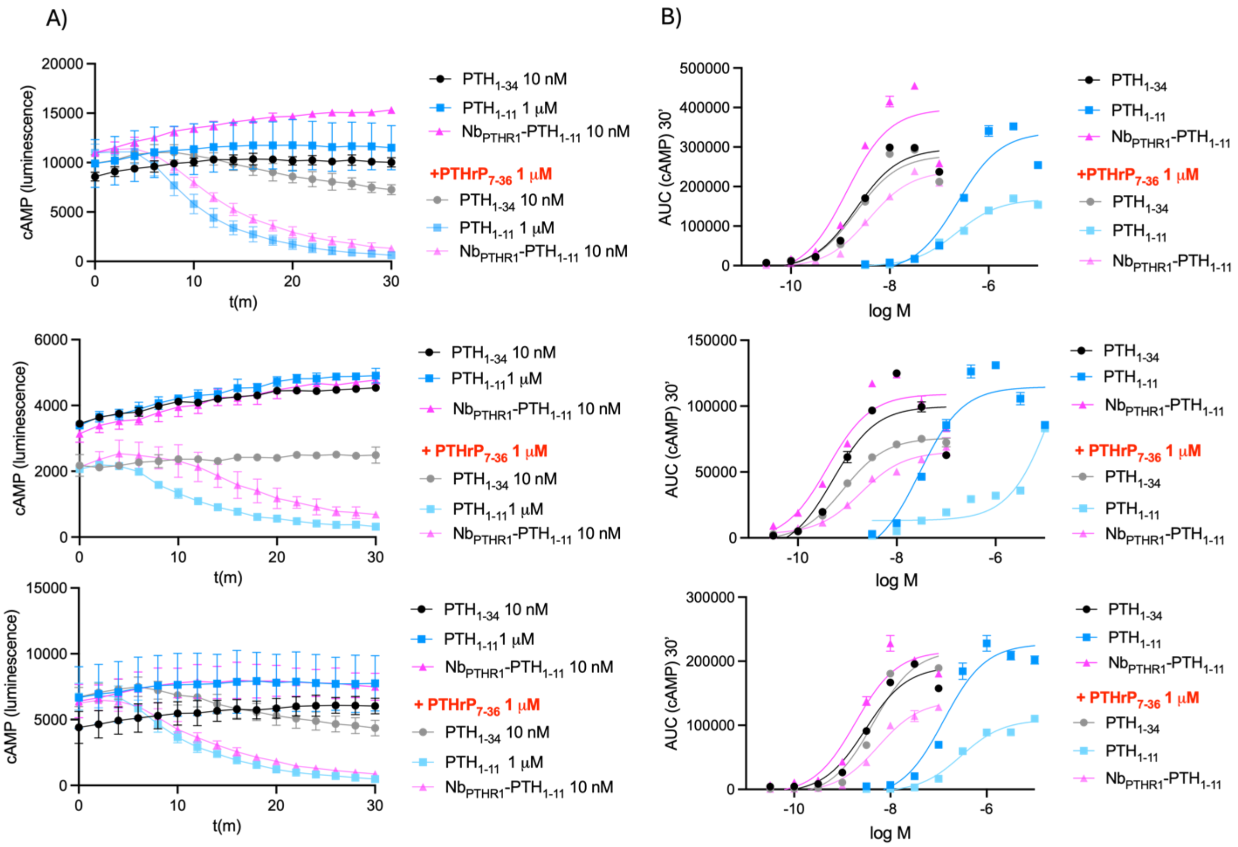

C)

Ligands	cAMP, Washout AUC			
	Control		+PTHrP$_{7-36}$	
	EC_{50} (±SEM), nM	% PTH$_{1-34}$ activation E_{max} (±SEM)	EC_{50} (±SEM), nM	% PTH$_{1-34}$ activation E_{max} (±SEM)
PTH$_{1-34}$	1.8 (0.7)	100	2.4 (1)	100
PTH$_{1-11}$	131 (59)	115 (1)	223 (41)	52 (4)
PTH$_{1-11}$-Nb$_{PTHR1}$	1.2 (0.4)	118 (7)	3.7 (1.2)	79 (7)

Supplementary Figure 18: **Evaluation of the impact of antagonist addition on cAMP signaling washout kinetics.** Washout responses were measured following the removal of free ligand as described in Methods. A) Representative time course of cAMP levels following ligand removal, either with or without an antagonist added during washout (PTHrP$_{7-36}$). B) Representative concentration-response curves for cAMP signaling ("washout") in the 30 minutes following ligand removal in the presence or absence of antagonist (PTHrP$_{7-36}$). Washout assay data were quantified as area under the curve from measurements performed every 2 minutes. The middle and bottom panel corresponds to independent experiments. Data points represent the mean ± SD from measurements performed as technical duplicates in an individual representative experiment. C) Compiled tabulation of ligand potency and E_{max} parameters derived from integrated area under the curve values from washout experiments. Indicated parameters correspond to mean ± SEM from three independent replicate experiments.

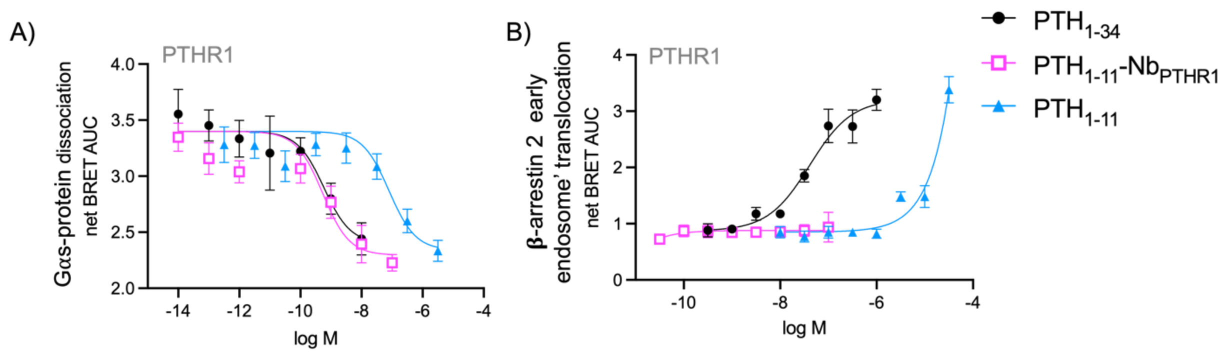

Supplementary Figure 19: Characterization of Nb-ligand conjugate signaling at WT PTHR1 through diverse pathways. Concentration-response curves for ligands were measured for the A) dissociation of Gαs protein, and B) recruitment of β-arrestin 2 to endosomes in HEK cells expressing WT PTHR1. All responses are presented as AUC generated from kinetic BRET measurements. Data points correspond to mean ± SD from technical replicates in a representative experiment. Curves are fit to a three-parameter logistic sigmoidal model. Tabulation of agonist potency parameters are show in main Table 1, derived from 3-5 independent experiments (see Supplementary Table 3).

Ligand	G protein				Arrestin				Ligand bias	
	Emax (%)	EC_{50} (nM)	Log ($Emax/EC_{50}$)	ΔLog ($Emax/EC_{50}$)	Emax (%)	EC_{50} (nM)	Log ($Emax/EC_{50}$)	ΔLog ($Emax/EC_{50}$)	ΔΔLog ($Emax/EC_{50}$)	Bias factor
PTH1-34	100	6.1	8.2	0.0 (ref.)	100	5.2	8.3	0.0 (ref.)	0.0	1.0
Nb_{PTHR1}-PTH1-11	100	9.2	8.0	-0.2	100	1000000	3.0	-5.3	5.1	127000
PTH1-11	100	103	7.0	-1.2	92	17230	4.7	-3.6	2.3	213.3
$Nb_{PTHR1X2}$-PTH-11	100	8.2	8.1	-0.1	100	1000000	3.0	-5.3	5.2	143000

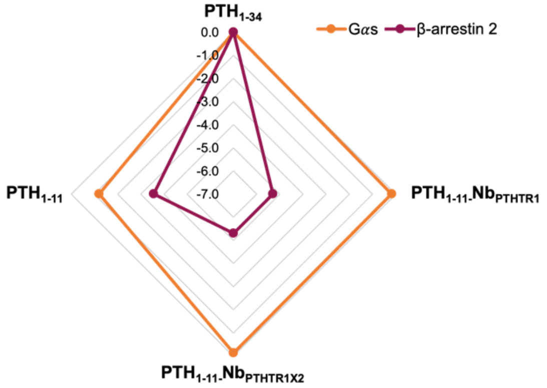

Supplementary Figure 20: Quantified bias factors for ligand-conjugates across functional assays. Potency (EC_{50}) and efficacy (Emax, percent of PTH_{1-34}) values for Gαs-cAMP and β-arrestin 2 recruitment for PTHR1 were generated from compiled data fit to three parameter logistic equation. Since the Nb-conjugates were virtually inactive in the arrestin recruitment assay, top and bottom parameters were constrained to match those of PTH_{1-34} to provide EC_{50} values for ligand bias calculations. ΔLog($Emax/EC_{50}$) values were calculated relative to PTH_{1-34} within each individual experiment. ΔΔLog($Emax/EC_{50}$) were then calculated between the indicated assays as described in Methods[2]. Bias factors were plotted as radar plot. The radar plot is based on the agonist type plotted against G protein and β-arrestin signaling. The values plotted correspond to ΔLog($Emax/EC_{50}$) values for each pathway.

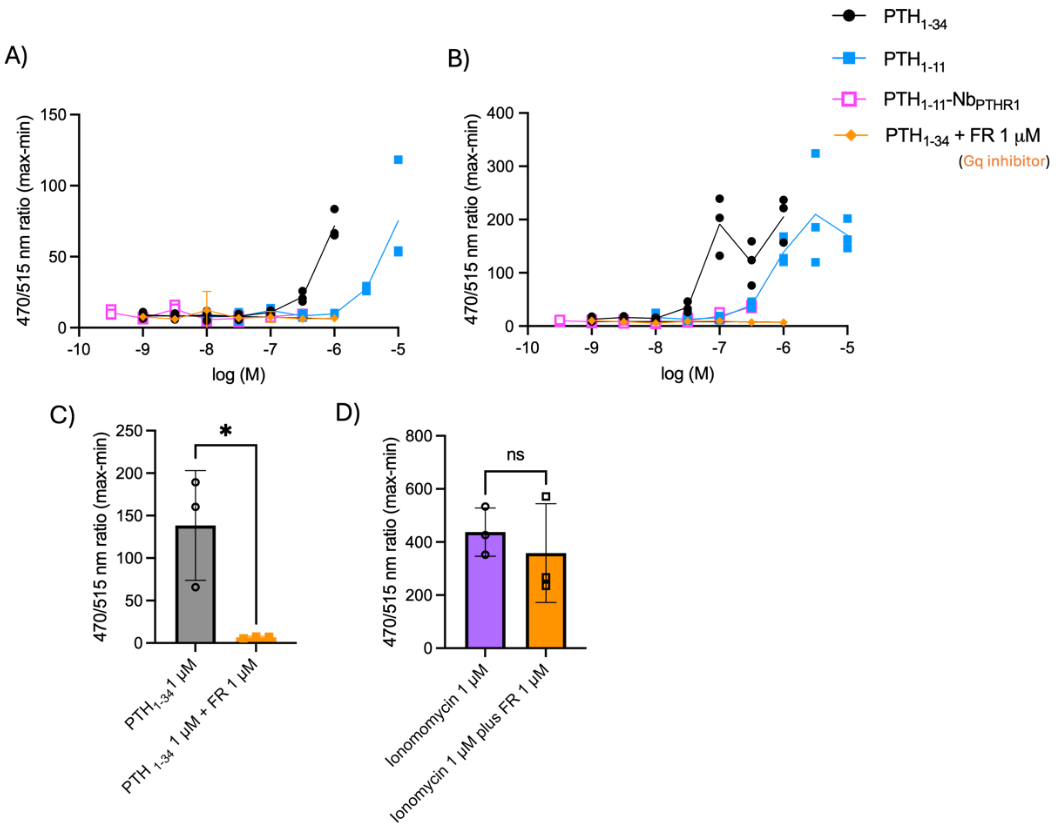

Supplementary Figure 21: **Evaluation of calcium mobilization by PTHR1 ligands and inhibition by an antagonist of Gαq signaling (FR).** Panels A + B correspond to independent experiments measuring calcium mobilization induced by PTHR1 ligands, as described in Methods. Individual data points (3 technical replicates per condition) correspond to responses recorded in individual wells with HEK-PTHR1 cells loaded with Calbryte 520 AM Ca^{2+} indicator dye. Lines connecting data mean values are shown only to guide the eye. C-D) Analysis of the impact of Gαq inhibitor FR900359 (FR, 1 μM) on PTH_{1-34} or ionomycin-mediated calcium mobilization. Cells were pre-treated with a Gαq inhibitor FR900359 (FR, 1 μM) before stimulation with PTH_{1-34} or ionomycin. Results are expressed as relative fluorescence units normalized to signal background. The bar graph corresponds to mean ± SD from three independent experiments. Statistical significance was assessed using an unpaired t-test ns not statistically significant; * $p = 0.0242$ for PTH_{1-34} versus PTH_{1-34} + FR.

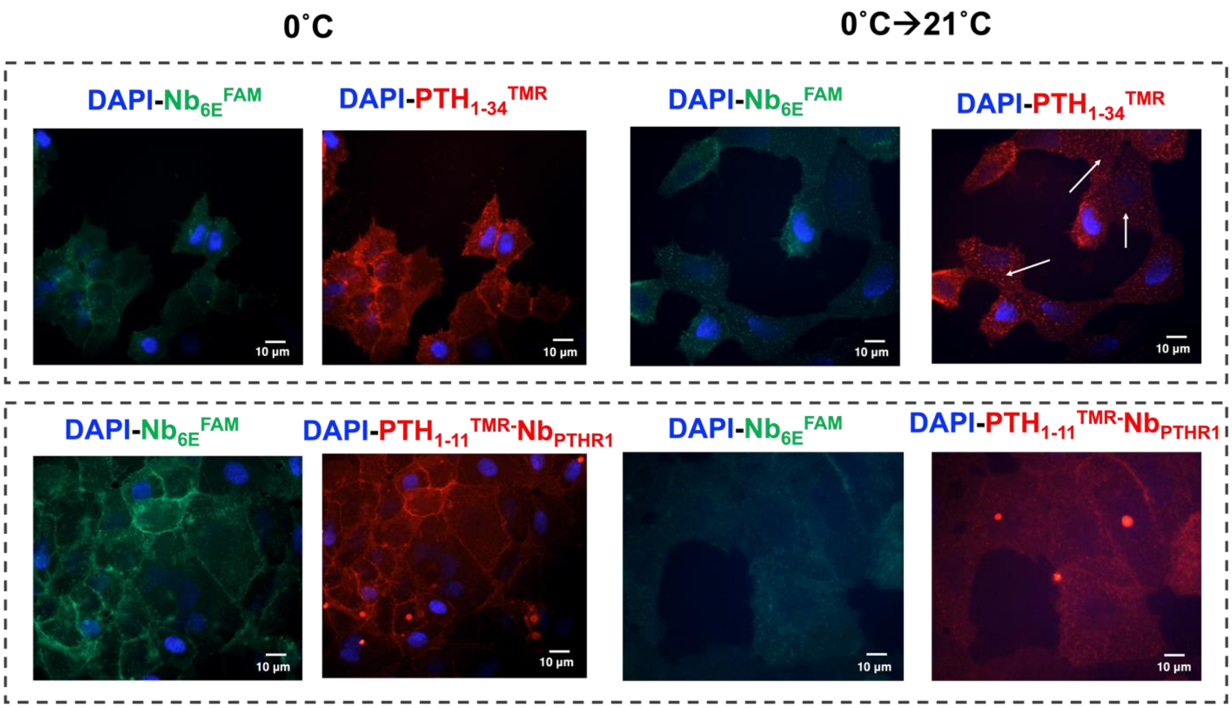

Supplementary Figure 22: Fluorescence microscopy analysis of ligand-induced internalization of PTHR1-6E. HEK cells expressing PTHR1-6E were visualized through staining with Nb_{6E}^{FAM} (green), DAPI (blue), and TMR-labeled ligand (red). Shown are representative images acquired after 30 min of stimulation with either 300 nM of $PTH_{1\text{-}34}^{TMR}$ (top) or $PTH_{1\text{-}11}$-Nb_{PTHR1}^{TMR} (bottom). $PTH_{1\text{-}11}$-Nb_{PTHR1}^{TMR} was synthesized from $PTH_{1\text{-}11}$-Cys(TMR)-Ahx-Azide and Nb_{PTHR1}-DBCO. Staining was performed on ice and incubation was carried out at either at 0°C (left) or 21°C (right). Following incubation cells were exposed to fixative prior to imaging. Scale bars, 10 µm. White arrows indicate punctate signals corresponding to ligand-induced intracellular accumulation of receptors. Data are representative of two replicate experiments.

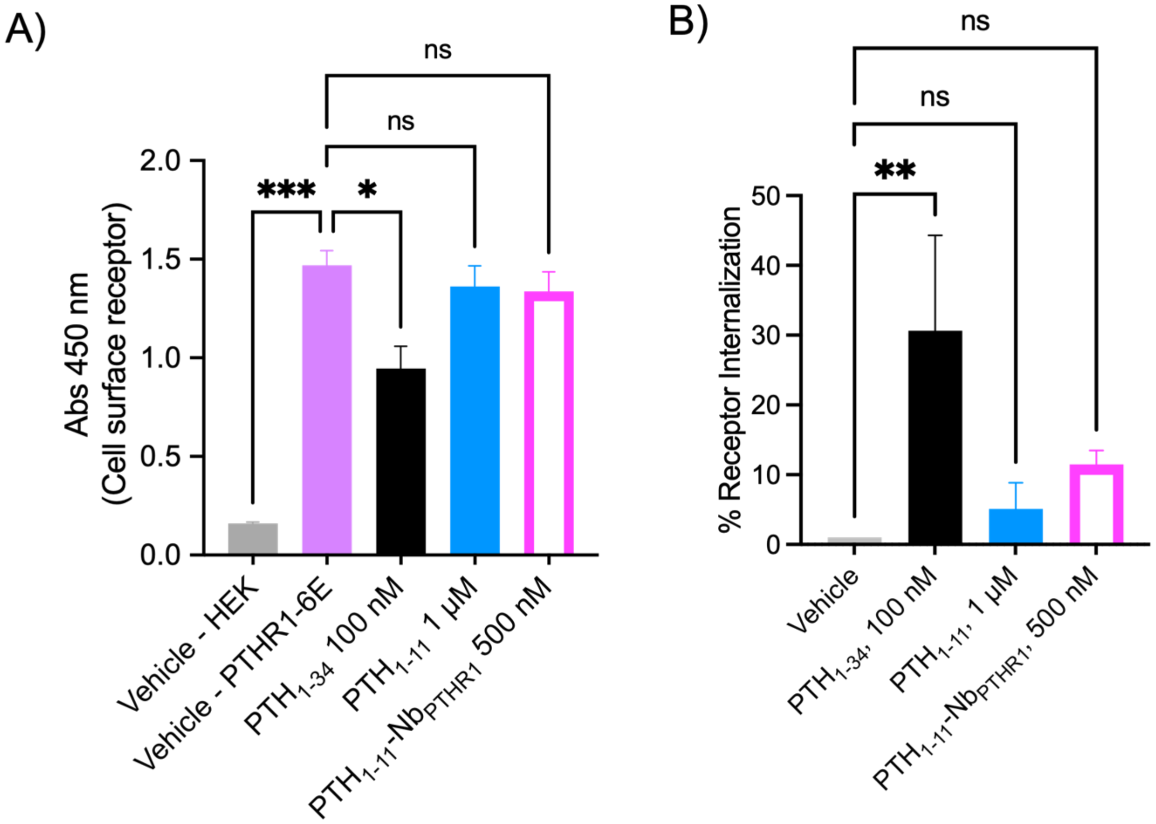

Supplementary Figure 23: Whole-cell ELISA to measure changes in surface levels of PTHR1-6E upon ligand exposure. A) Intact, adherent cells expressing PTHR1-6E were exposed to ligand or vehicle at 37°C. After incubation, cell surface receptors were detected with Nb_{6E}-biotin and secondary staining with horseradish peroxidase-conjugated streptavidin. A) Representative data showing signal from detection of cell surface receptor following 30 minutes of treatment with indicated ligands. “Vehicle-HEK” indicates the use of cells not expressing PTHR1-6E and without exposure to ligands. “Vehicle-PTHR1-6E” indicates the use of cells expressing PTHR1-6E without exposure ligands. B) Graphic showing receptor internalization, quantified as the percent loss of cell surface receptors in ligand-treated cells normalized to Vehicle-HEK (negative control cells). Data points correspond to mean ± SEM from 3 independent experiments conducted with technical replicates in each independent experiment. Statistical significance was assessed by one-way ANOVA, with Dunnett’s post hoc correction (*$p < 0.05$; **$p < 0.01$; ***$p < 0.001$; ****$p < 0.0001$; ns not significant). For panel A, Vehicle-HEK versus Vehicle-PTHR1-6E P = 0.0004 and Vehicle-PTHR1-6E versus $PTH_{1\text{-}34}$ P = 0.015. For Panel B, Vehicle versus $PTH_{1\text{-}34}$ P = 0.0016.

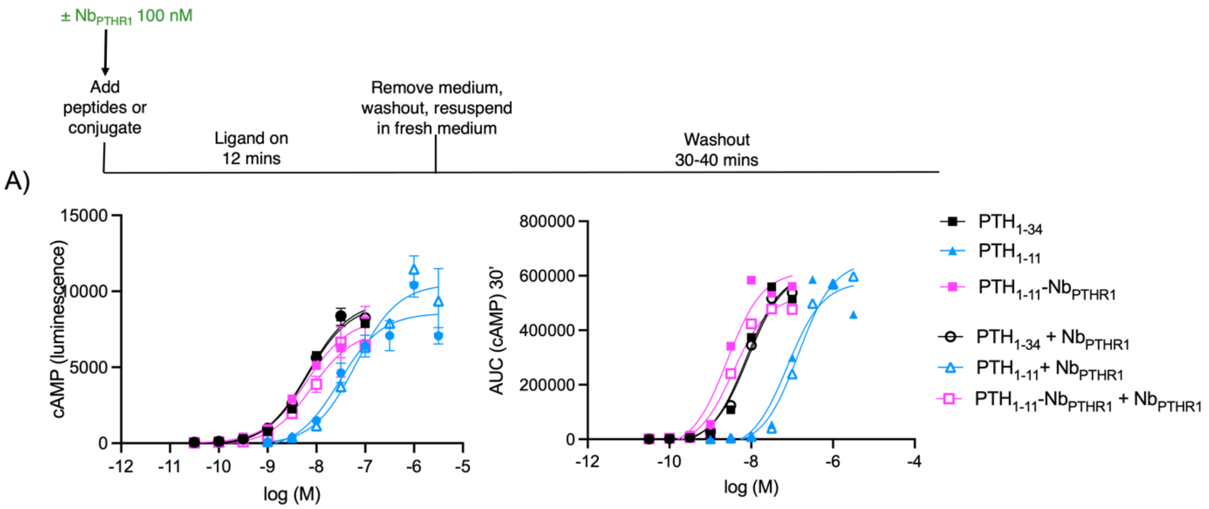

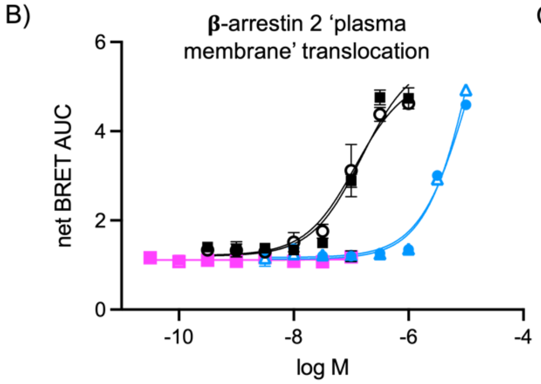

C)

PTHR1 EC50 (±SEM), nM w/o Nb_{PTHR1}	cAMP	Washout AUC	β-arrestin 2 (plasma membrane)
PTH_{1-34}	11 (7)	6.7 (1.4)	62 (44)
PTH_{1-11}	22 (11)	70 (7)	11000 (940)
PTH_{1-11}-Nb_{PTHR1}	2.8 (1)	2.4 (0.3)	-
**with Nb_{PTHR1}**			
$PTH1_{-34}$	15 (11)	5.3 (1.3)	54 (32)
PTH_{1-11}	32 (16)	115 (11)	12000 (1090)
PTH_{1-11}-Nb_{PTHR1}	3.5 (1.4)	3.7 (1.4)	-

Supplementary Figure 24: Evaluation of the effects of Nb_{PTHR1} co-administration on ligand signaling properties at PTHR1. A) Timeline for the addition of ligands and Nbs in the cAMP assay. Concentration-response data for ligand-conjugates for cAMP production were assessed on WT PTHR1 with or without Nb_{PTHR1} (100 nM). Data at right correspond to ligand washout responses generated from quantifying the area under the curve for kinetic signals following washout. Data points correspond to mean ± SD from technical replicates in a single representative experiment. Curves were generated using a three-parameter logistic sigmoidal model. B) Representative curve for stimulation of β-arrestin 2 recruitment to plasma membrane on WT PTHR1 with or without Nb_{PTHR1}. C) Tabulation of compiled agonist potency parameters for ligand-conjugates at PTHR1. EC_{50} values correspond to mean (±SEM) measurements from 3 biological replicates. Note that these data are distinct from those shown in Table 1 in the main text.

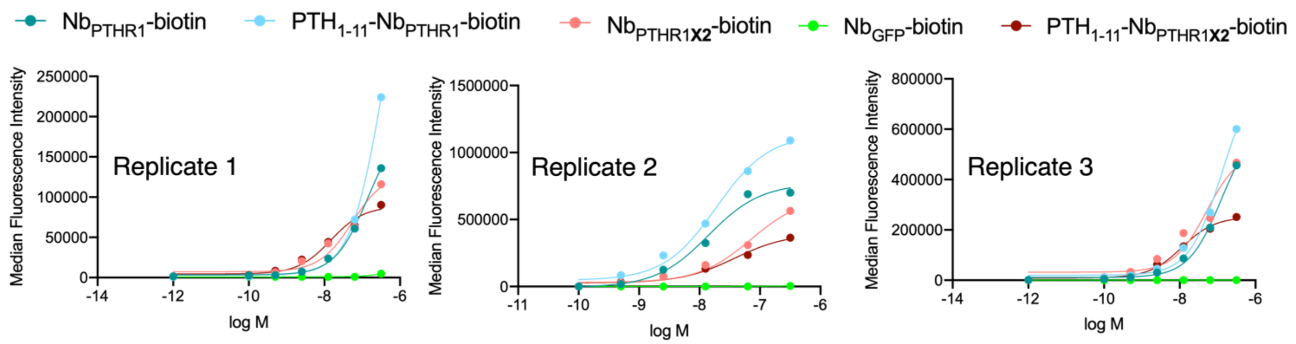

Supplementary Figure 25: Flow cytometry analysis comparing the binding of Nb and PTH$_{1-11}$-Nb conjugates to PTHR1. Varying concentration of Nbs and ligand-conjugates labeled with biotin via sortagging were incubated with cells expressing PTHR1 followed by washing, detection with streptavidin-APC, and assessment of cellular fluorescence. Nb$_{GFP}$ refers to negative control Nb that binds to GFP not present in this receptor construct[3]. Each graphic corresponds to an independent replicate. Connecting lines were not used for EC_{50} calculations and only serve to guide the eye.

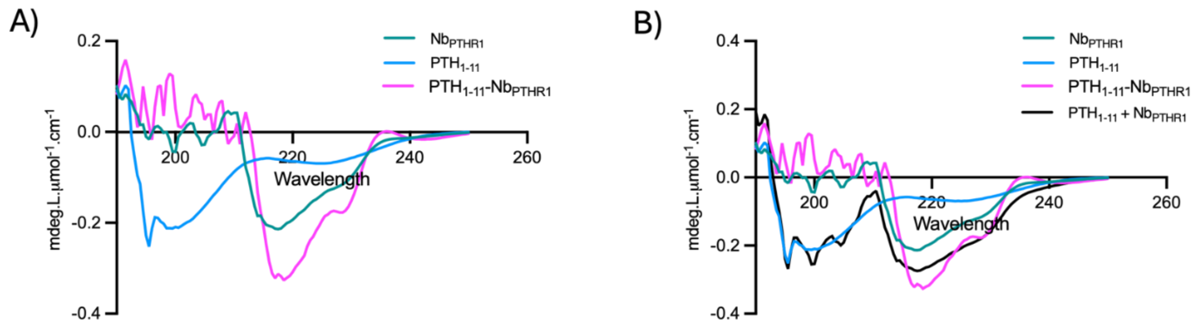

Supplementary Figure 26: Circular dichroism (CD) analysis of ligand conformational properties. A) Far UV-CD spectra for Nb$_{PTHR1}$, PTH$_{1-11}$, and Nb$_{PTHR1}$-PTH$_{1-11}$ measured at 25°C in TBS buffer. These spectra have been blank corrected. B) Comparison of the sum of the Nb$_{PTHR1}$ and PTH$_{1-11}$ spectra (shown in black) to experimentally recorded spectra.

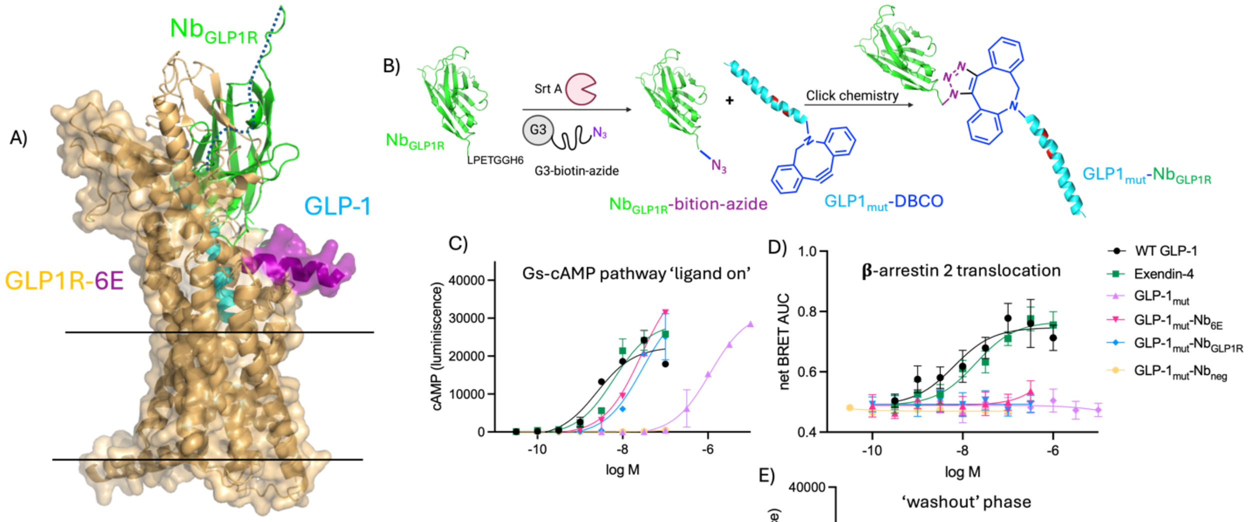

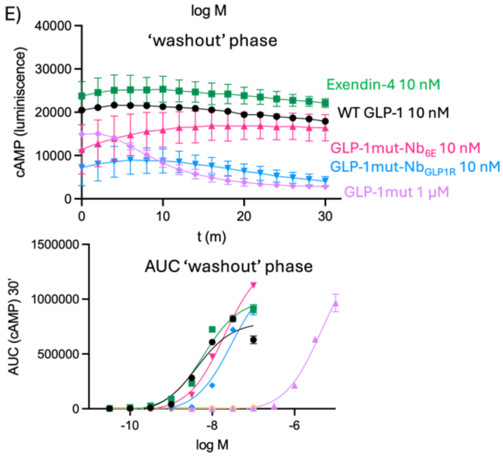

F)

EC50 (±SEM), nM Ligands	cAMP	Washout AUC	β-arrestin 2
WT GLP-1	5 (2)	5 (0.5)	40 (15)
Exendin-4	5 (1.4)	3.7 (2)	33 (4)
GLP-1_{mut}	1616 (600)	6475 (2000)	-
GLP-1_{mut}-Nb_{6E}	15 (6)	10 (4.5)	-
GLP-1_{mut}-Nb_{GLP1R}	43 (12)	42 (16)	-
GLP-1_{mut}-Nb_{neg}	-	-	-

	Sequence
WT GLP-1	HAEGTFTSDVSSYLEGQAAKEFIAWLVKGRG
GLP-1_{mut} (F22A/L26A GLP-1)	HAEGTFTSDVSSYLEGQAAKEAIAWAVKGRGC

Supplementary Figure 27: **Assessment of peptide and Nb-ligand conjugate activation of GLP1R**. A) A model of GLP1R with a 6E epitope tag appended to its N-terminus (GLP1R-6E, see Supplementary Methods for sequence) bound to a previously described[4] GLP1R-binding Nb was generated using Alphafold2 (see Methods) and overlaid with a previously published cryo-EM structure of GLP1R (PDB: 6VCB) bound to ligand. GLP1R-6E (in wheat with 6E tag in purple) is shown bound to Nb_{GLP1R} and GLP-1 (green and cyan). The dark black dashed lines depict a hypothetical linker between the C-terminus of Nb_{GLP1R} and GLP-1 peptide in these models. Note that this model is for illustration purposes only and is not intended to suggest a specific site for Nb_{GLP1R} binding. B) Synthetic scheme for GLP-1 ligand-Nb conjugate preparation via the azide-alkyne cycloaddition chemistry. GLP-1_{mut} corresponds to a double variant of GLP1 with Ala mutations at positions known to be important for the binding of this peptide to its receptor[5] (see panel F for sequence, with mutations highlighted in red font). C-E) Measurement of the signaling properties of prototype GLP1R peptide ligands and Nb-peptide conjugates. Ligand-induced C) cAMP responses and D) β-arrestin2 recruitment to the plasma membrane were measured in HEK cells transfected and selected to stably express GLP1R-6E, as described in Methods. E) GLP1R ligand washout studies were performed using the same protocol used for PTHR1 (see Methods). Washout responses were quantified via measurement of area under the curve (AUC) from time course plots of cAMP levels following ligand removal. (Left) Concentration-response curves are shown are fit to a three-parameter logistic sigmoidal model. (Right) Representative kinetic traces of washout responses of single concentrations for all ligands. Individual data points correspond to mean ± SD from technical replicates in a single representative experiment F) Compiled tabulation of agonist potency parameters for ligand-conjugates at GLP1R-6E. EC_{50} values

correspond to mean (±SEM) measurements from 3 independent biological replicates. A dash indicates that activity was too weak to calculate an EC_{50} value.

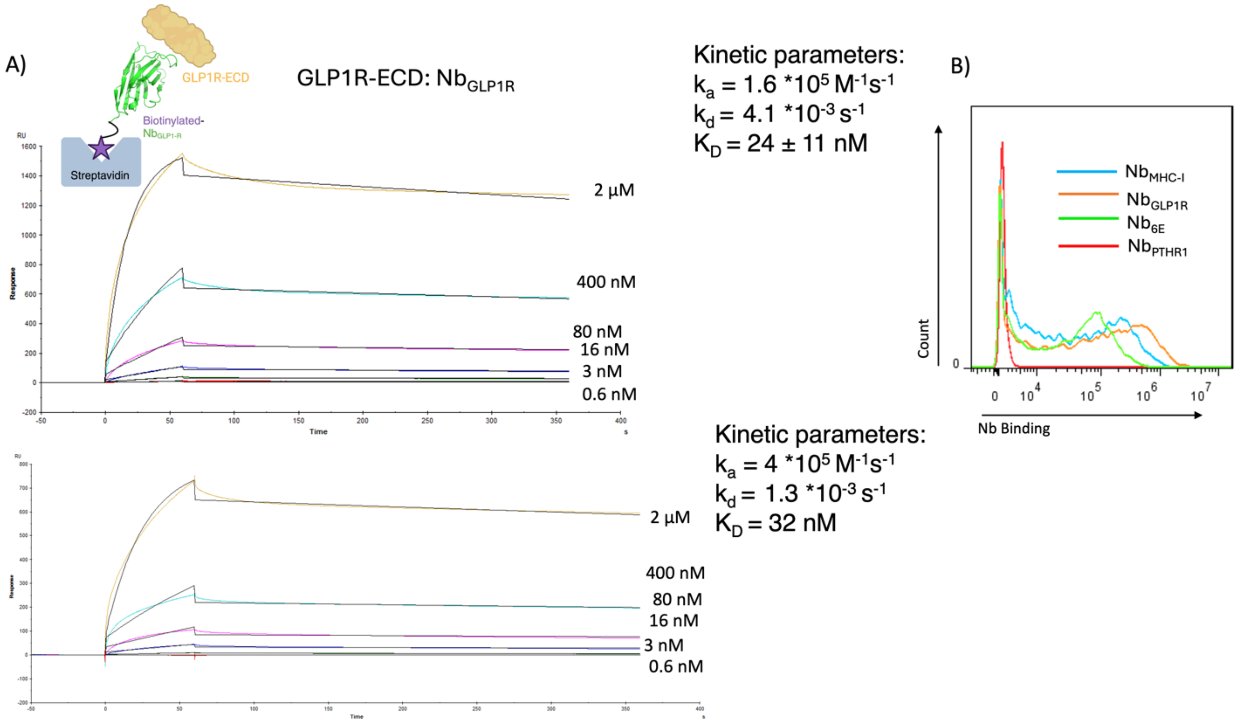

Supplementary Figure 28: Assessment of Nb binding to GLP1R. A) Independent replicate for the measurement of GLP-1 and Nb_{GLP1R}[6] binding by surface plasmon resonance. The composition of GLP1R-ECD protein is described in Supplementary Figure 15 . Nb_{GLP1R} was conjugated to biotin at its C-terminus using sortagging and immobilized onto sensor chips functionalized with streptavidin as described in Methods. The association phase lasted for 60 s followed by an addition 300 s of dissociation. k_a and k_d are the association and dissociation rate constants, and K_D is the dissociation constant. KD values correspond to mean ± SD from measurements performed with different concentrations of GLP1R-ECD. B) Representative histograms for flow cytometry analysis of Nb_{GLP1R} (orange) and Nb_{PTHR1} (red) binding to cells stably expressing GLP1R-6E (See Supplementary Methods). Nbs (300 nM) labeled with biotin were incubated with cells expressing GLP1R-6E, followed by washing, detection with streptavidin-APC, and assessment of cellular fluorescence. Panel A was created in part with BioRender.com released under a Creative Commons Attribution-NonCommercial-NoDerivs 4.0 International license: https://creativecommons.org/licenses/by-nc-nd/4.0/deed.en

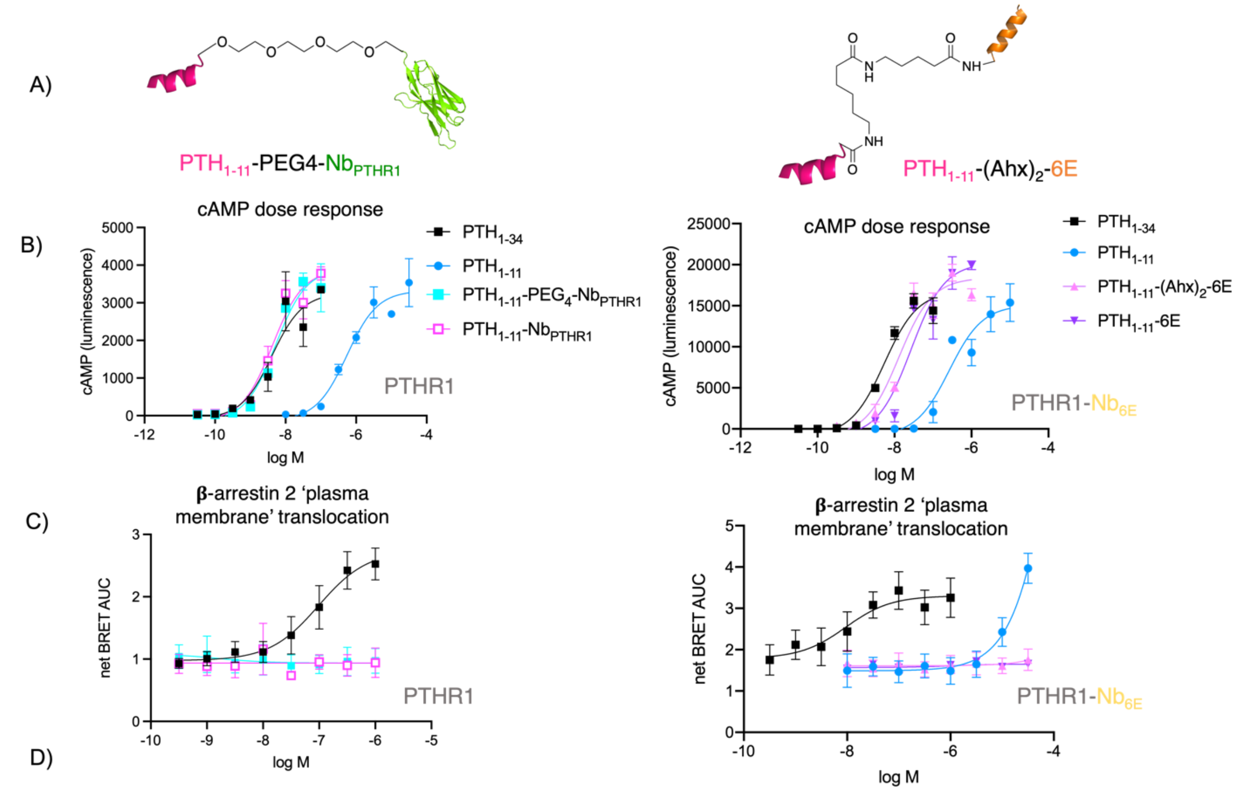

D)

Ligands EC50 (±SEM), nM PTHR1	cAMP	β-arrestin 2 (plasma membrane)
PTH_{1-34}	3.3 (3)	87 (47)
PTH_{1-11}	370 (160)	ND
PTH_{1-11}-PEG_4-Nb_{PTHR1}	2.4 (1)	-
PTH_{1-11}-Nb_{PTHR1}	18 (15)	-

Ligands EC50 (±SEM), nM PTHR1-Nb_{6E}	cAMP	β-arrestin 2 (plasma membrane)
PTH_{1-34}	3.1 (1.2)	26 (10)
PTH_{1-11}	140 (64)	11000 (6500)
PTH_{1-11}-$(Ahx)_2$-6E	9.4 (3)	-
PTH_{1-11}-6E	12 (6)	-

Supplementary Figure 29: Impact of longer linker length conjugates signaling activity. A) Schematic representation of the conjugates with longer linkers used for experiments in this Figure. Compounds were synthesized as described in the methods section. Concentration-response curves for B) cAMP production, and C) β-arrestin 2 recruitment in HEK cells expressing PTHR1 (left) and PTHR1-Nb_{6E} (right). D) Compiled tabulation of agonist potency parameters for conjugates with longer linkers at PTHR1 and PTHR1-Nb_{6E}. EC_{50} values correspond to mean (±SEM) measurements from 3 independent experiments. "ND" indicates that the response was not measured in these assays. Note that these data are distinct from those in Table 1 in the main text.

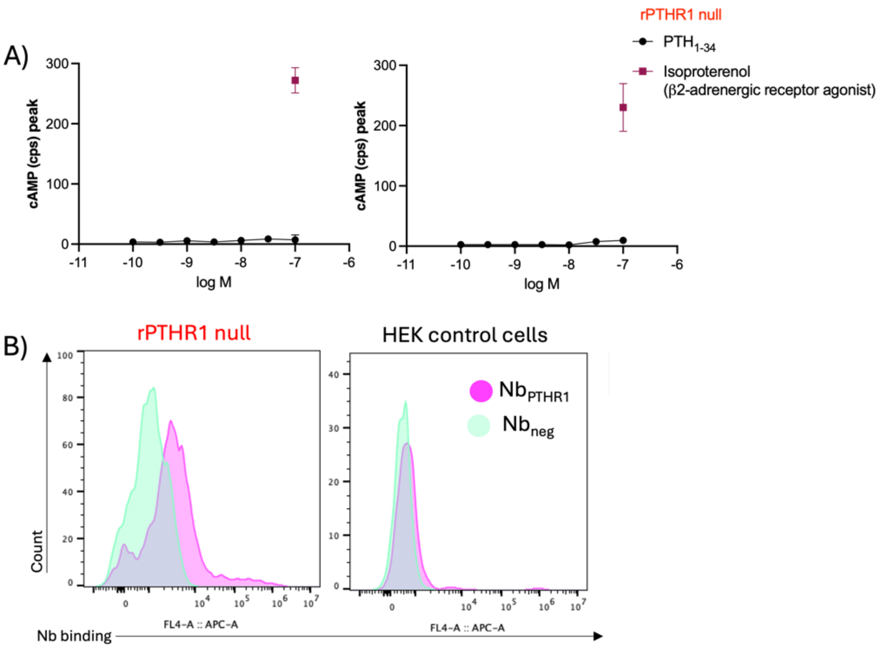

Supplementary Figure 30: Lack of signaling activity of rPTHR1 null in response to $PTH_{1\text{-}34}$. A) Representative concentration-response curves for the induction of cAMP responses by $PTH_{1\text{-}34}$ in cells stably expressing cAMP-responsive luciferase (Glosensor) transiently transfected with rPTHR1 null receptor (rat PTHR1 R233Q/Q451K) using transfection protocols described in Methods. Isoproterenol was used as a positive control to induce a cAMP response through β2-adrenergic receptor, which is known to be endogenously expressed in HEK cells[7]. Each graph corresponds to an independent biological replicate. Data points correspond to mean ± SD from technical duplicates. B) Flow cytometry analysis of Nb_{PTHR1} or Nb_{neg} binding to the HEK cells transiently transfected rPTHR1 null or non-transfected comparator (HEK control) cells. Cells were treated with Nb-biotin (300 nM), followed by staining with streptavidin-APC, as described in Methods. Data is presented as a histogram of staining of live cells in the APC (FL4) channel.

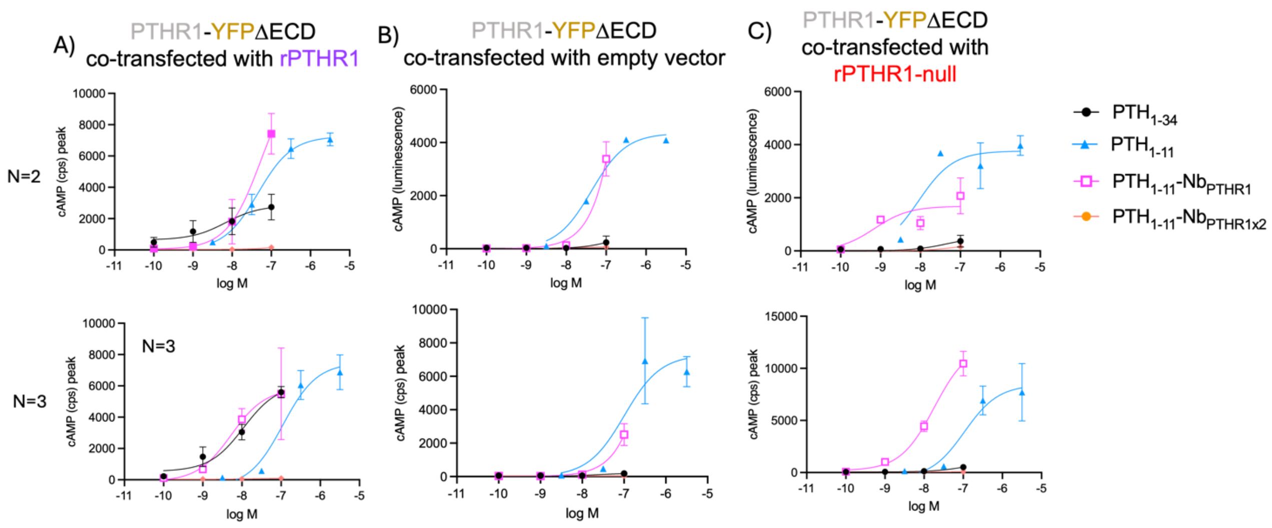

Supplementary Figure 31: Independent experiments for receptor 'activation in trans'. Dose response curves for ligand conjugates for induction of cAMP responses in cells co-transfected with PTHR1-YFPΔECD (PTHR1 ECD replaced with YFP) and A) rPTHR1, B) empty vector, or C) rat-PTHR1 null. Curves were generated by fitting to a three-parameter logistic equation (separate from that shown in main Figure 6).

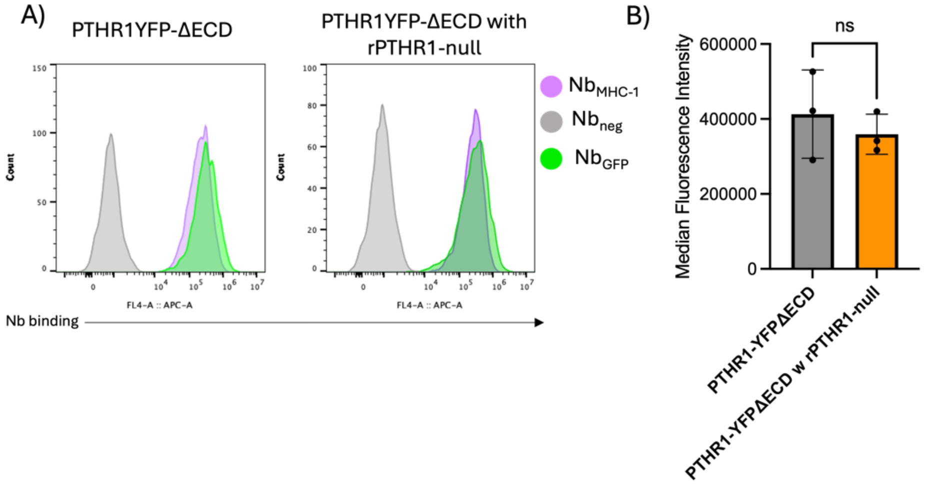

Supplementary Figure 32: Effect of co-expression of rPTHR1-null on PTHR1YFP-ΔECD expression levels. A) Flow cytometry analysis of HEK cell line stably expressing PTHR1YFP-ΔECD alone or the same cell line transiently transfected with rPTHR1-null receptor. Cells were labeled with Nb-biotin conjugates followed by detection with streptavidin-APC, as described in methods. $Nb_{MHC\text{-}I}$ is included for comparison to the staining intensity observed with a highly expressed cell surface protein. Data is presented as a representative histogram of staining of live cells in the APC (FL4) channel. B) The intensity of cellular staining was measured through quantitation of median fluorescence intensity (MFI) values. MFI values are derived from APC histograms as shown in panel A. Data points and error bars correspond to mean (MFI) ± SD from three independent biological replicates. Statistical significance was assessed using an unpaired t-test (ns not statistically significant).

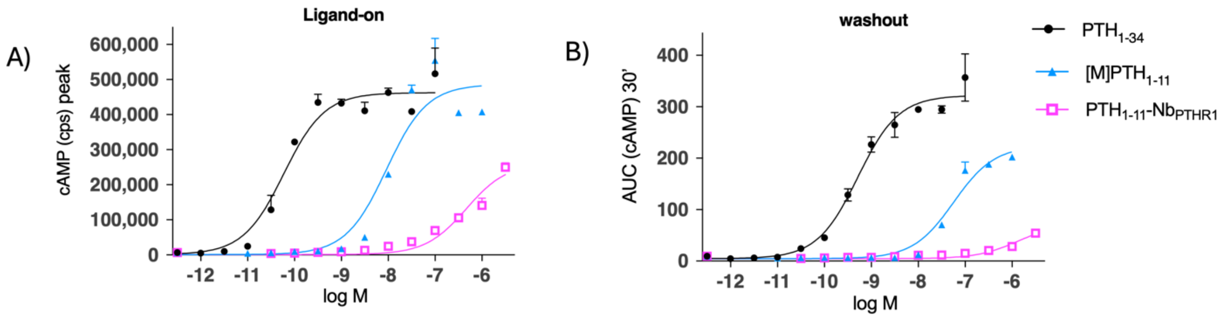

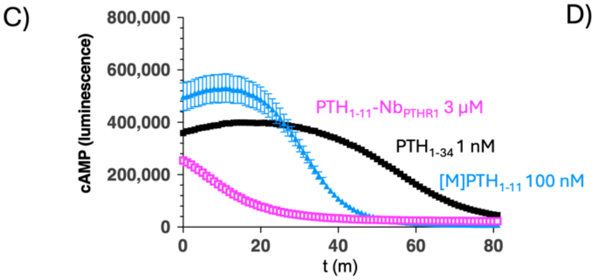

D)

EC_{50} (±SD), nM Ligands	cAMP	Washout AUC
PTH_{1-34}	0.05 (0.03)	0.3 (0.2)
$[M]PTH_{1-11}$	6 (4)	50 (35)
PTH_{1-11}-Nb_{PTHR1}	>10000	-

Supplementary Figure 33: Characterization of PTHR1 ligands and conjugates on cells expressing endogenous levels of PTHR1. PTHR1-ligand meditated cAMP production was monitored using a SaOS2-derived cell line (SGS72)[8] that expresses PTHR1 at endogenous levels and is stably transfected to express a cAMP-activated variant of luciferase[9]. Due to low levels of receptor expression, the protocol for these experiments was different than those performed with HEK293 cells (see Supplementary Methods). A) Representative concentration-response curve of ligand-induced cAMP production in SGS72 cells. Data points correspond maximal luminescence (mean ± SD) responses observed 12-20 minutes after ligand addition. B) Representative concentration-response curve from quantifying the area under the curve of time course washout responses. Curves in panels A + B were generated by fitting data to a 3-parameter sigmoidal dose-response model. C) Representative kinetic washout trace from washout of ligands from SaOS2 cells at indicated doses. Data points in panels A-C correspond to mean ± SD. D) Compiled tabulation of agonist potency parameters for ligand-conjugates on SGS72 cells. EC_{50} values correspond to mean (±SD) measurements from 3 independent biological replicates. The sequence of [M]PTH1-11 is available in Supplementary Figure 35.

>Nb_{PTHR1}
EVQLVESGGG LVQAGGSLRL SCAASGNIFA NNIMGWYRQP PGKEREFVAH VSHDGDSMYA
VSVKGRFAIS RKDATNLYLQ MNSLKPEDTA IYFCRLLNIP TQGRMEGFWG QGTQVTVSS
>$Nb_{PTHR1\text{-}\mathbf{X2}}$
EVQLVESGGG LVQAGGSLRL SCAASGLTFS NYAMGWFRQA PGKEREWVAS INWSGGSTYY
EDSVEGRFTI SRDNAKNTVN LQMNSLKPED TAVYYCAAKR GHYSREYDYW GQGTQVTVSS
>Nb_{6E}
QVQLQE SGGGLVQPGG SLRLSCAASG FVFENSAMAW YRQAPGKERE LIAVIGTTFI
KLAESVKGRF TISRDNAKST VYLQMNNLKP EDTAVYYCSK SGAYWGQGTQ VTVSS
>Nb_{GLP1R}
EVQLVESGGG LVQAGGSLRL SCAASGNTYS YKVMGWFRQA PGKEREFVGI IIRNGDTTYY
ADSVKGRFTI SADNAKNTVY LQMNSLKPED TAVYYCAASP KYMTAYERSY DWGQGTQVTV
SS

```
NbPTHR1         EVQLVESGGGLVQAGGSLRLSCAASGNIFANNIMGWYRQPPGKEREFVAHVSHDGDS-MY      59
Nb6E            QVQLQESGGGLVQPGGSLRLSCAASGFVFENSAMAWYRQAPGKERELIAVIGT--TFIKL      58
NbPTHR1-X2      EVQLVESGGGLVQAGGSLRLSCAASGLTFSNYAMGWFRQAPGKEREWVASINWSGGSTYY      60
NbGLP1R         EVQLVESGGGLVQAGGSLRLSCAASGNTYSYKVMGWFRQAPGKEREFVGIIIRNGDTTYY      60
                :*** ******** ************  :    *.*:** ****** :. :

NbPTHR1         AVSVKGRFAISRKDAT-NLYLQMNSLKPEDTAIYFCRLLN--IPTQGRMEGFWGQGTQVT      116
Nb6E            AESVKGRFTISRDNAKSTVYLQMNNLKPEDTAVYYCSKSG----------AYWGQGTQVT      108
NbPTHR1-X2      EDSVEGRFTISRDNAKNTVNLQMNSLKPEDTAVYYCAAKRGH---YSREYDYWGQGTQVT      117
NbGLP1R         ADSVKGRFTISADNAKNTVYLQMNSLKPEDTAVYYCAASPKYMTAYERSY-DWGQGTQVT      119
                  **:***:** .:*. .: ****.*******:*:*                ********

NbPTHR1         VSS   119
Nb6E            VSS   111
NbPTHR1-X2      VSS   120
NbGLP1R         VSS   122
                ***
```

Supplementary Figure 34: Sequence information and annotated sequence alignment data for Nbs used in this study. Alignment was performed using ClustalOmega using the entire sequence for each Nb. "*" indicates an exact match, ":" indicates a strong match, "." indicates a weak match. Amino acids are numbered according to their position within the sequence.

A)

Peptide	Sequence
$PTH_{1\text{-}28}$	UVUEIQLMHQhRAKWLNSMRRVEWLRKKL
$PTH_{1\text{-}21}$	AVUEIQLMHQhRAKWLNSMRRV
$[M]PTH_{1\text{-}11}$	ACPCVUEIQLMHQhR
$PTHrP_{7\text{-}36}$	LLHDLdWKSIQDLRRRFWLHHLIAEIHTAEY

B)

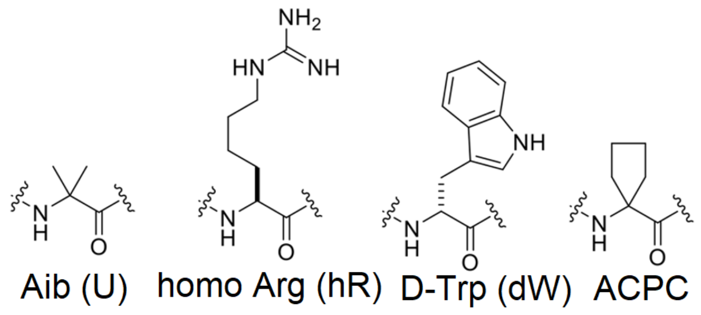

Supplementary Figure 35: **Sequences of selected peptides used in competition binding assays.** A) Colored lettering indicates different unnatural amino acids in the peptide. B) Structures of amino acid residues presented in colored lettering.

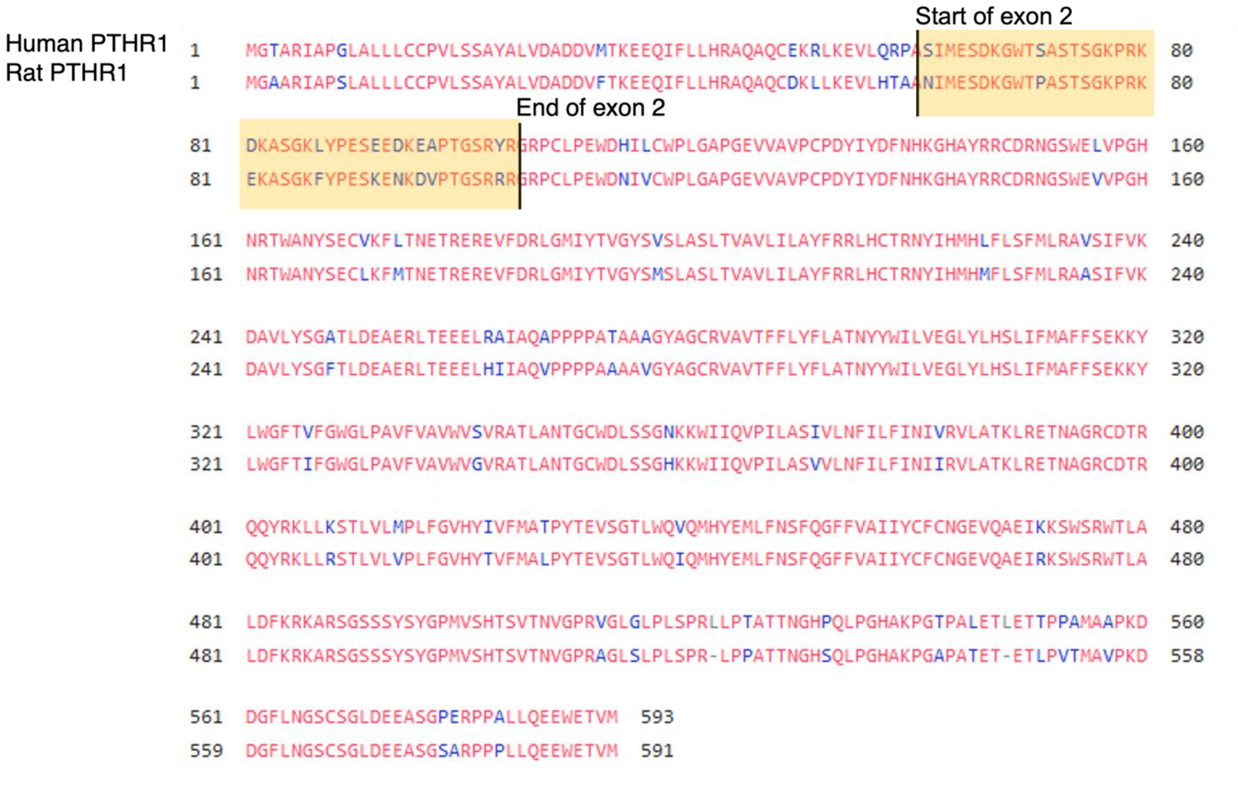

Supplementary Figure 36: Amino acid alignment of human and rat PTHR1. The sequence from exon 2 is highlighted in yellow. Red lettering indicates conservation. Blue lettering indicates no conservation. Alignment was performed using ClustalOmega. Amino acids are numbered according to their position within the sequence.

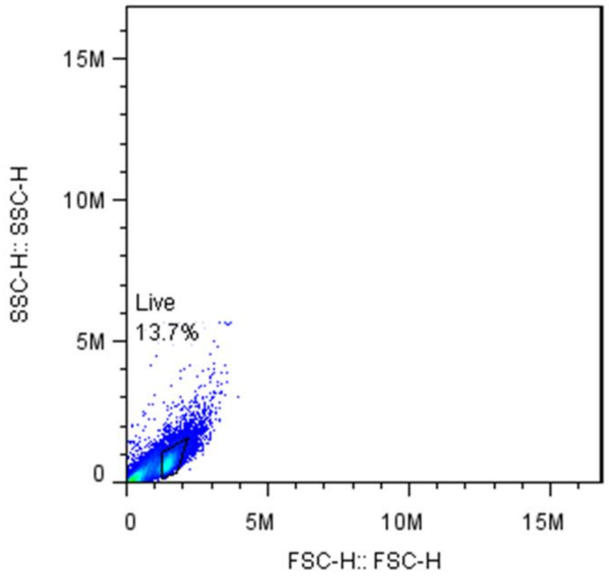

Supplementary Figure 37: Representative gating strategy for flow cytometry analyses. Samples were analyzed as described in Methods. The forward scatter side scatter gating strategy below was used to select intact, typical cells for further analysis of staining with fluorescently labeled compounds.

<u>**Supplementary Methods**</u>:

Full length GLP1R expression vector. A custom plasmid encoding human GLP1R with a 6E epitope tag (purple and underlined) inserted after the signal sequence near the protein N-terminus (sequence below) was produced commercially by VectorBuilder. The plasmid included elements encoding neomycin (G418) resistance and a CAG promoter for the protein of interest. This plasmid was transfected into a HEK293-based cell line (GS22, ref. 7) that stably expresses the cAMP biosensor Glosensor (22F). Limiting dilution in the presence of antibiotic (G418, 0.5 mg/mL) was performed to generate a clonal cell line that stably expresses both Glosensor and GLP1R-6E. This cell line was used for both cAMP and beta-arrestin recruitment assays, performed as described in the main Methods, to assess activation of GLP1R.

GLP1R-6E

Amino acid

MAGAPGPLRLALLLLGMVGRAGPRPQ<u>QADQEAKELARQIS</u>GATVSLWETVQKWREYRRQCQ
RSLTEDPPPATDLFCNRTFDEYACWPDGEPGSFVNVSCPWYLPWASSVPQGHVYRFCTAEGL
WLQKDNSSLPWRDLSECEESKRGERSSPEEQLLFLYIIYTVGYALSFSALVIASAILLGFRHLHCT
RNYIHLNLFASFILRALSVFIKDAALKWMYSTAAQQHQWDGLLSYQDSLSCRLVFLLMQYCVAA
NYYWLLVEGVYLYTLLAFSVLSEQWIFRLYVSIGWGVPLLFVVPWGIVKYLYEDEGCWTRNSNM
NYWLIIRLPILFAIGVNFLIFVRVICIVVSKLKANLMCKTDIKCRLAKSTLTLIPLLGTHEVIFAFVMDE
HARGTLRFIKLFTELSFTSFQGLMVAILYCFVNNEVQLEFRKSWERWRLEHLHIQRDSSMKPLK
CPTSSLSSGATAGSSMYTATCQASCS*

Nucleotide

atggccggcgcccccggcccgctgcgccttgcgctgctgctgctcgggatggtgggcagggccggcccccgcccccagcaggctg
accaagaagcaaaagaattggcacggcaaatcagcggtgccactgtgtccctctgggagacggtgcagaaatggcgagaatacc

gacgccagtgccagcgctccctgactgaggatccacctcctgccacagacttgttctgcaaccggaccttcgatgaatacgcctgctg
gccagatggggagccaggctcgttcgtgaatgtcagctgcccctggtacctgccctgggccagcagtgtgccgcagggccacgtgta
ccggttctgcacagctgaaggcctctggctgcagaaggacaactccagcctgccctggagggacttgtcggagtgcgaggagtcca
agcgaggggagagaagctccccggaggagcagctcctgttcctctacatcatctacacggtgggctacgcactctccttctctgctctg
gttatcgcctctgcgatcctcctcggcttcagacacctgcactgcacccggaactacatccacctgaacctgtttgcatccttcatcctgcg
agcattgtccgtcttcatcaaggacgcagccctgaagtggatgtatagcacagccgcccagcagcaccagtgggatgggctcctctc
ctaccaggactctctgagctgccgcctggtgtttctgctcatgcagtactgtgtggcggccaattactactggctcttggtggagggcgtgt
acctgtacacactgctggccttctcggtcttatctgagcaatggatcttcaggctctacgtgagcataggctggggtgttcccctgctgtttgt
tgtcccctggggcattgtcaagtacctctatgaggacgagggctgctggaccaggaactccaacatgaactactggctcattatccggc
tgcccattctctttgccattggggtgaacttcctcatctttgttcgggtcatctgcatcgtggtatccaaactgaaggccaatctcatgtgcaa
gacagacatcaaatgcagacttgccaagtccacgctgacactcatcccccctgctggggactcatgaggtcatctttgcctttgtgatgga
cgagcacgcccggggggaccctgcgcttcatcaagctgtttacagagctctccttcacctccttccaggggctgatggtggccatcttata
ctgctttgtcaacaatgaggtccagctggaatttcggaagagctgggagcgctggcggcttgagcacttgcacatccagagggacag
cagcatgaagcccctcaagtgtcccaccagcagcctgagcagtggagccacggcgggcagcagcatgtacacagccacttgcca
ggcctcctgcagctga

Assessment of receptor trafficking using fluorescence microscopy
Trafficking of PTHR1-6E was visualized as previously described[7]. Visualization was performed using a Nikon Eclipse Ni confocal microscopy. Briefly, cells expressing PTHR1-6E were seeded on glass coverslips for 24h. Staining was performed in HBSS solution supplemented with 10 mM HEPES pH 7.4 and 0.1% BSA on ice. The cells were then incubated with peptide or Nb-PTH_{1-11} conjugates at 0 or 21°C for 30 min. Following washing, the cells were fixed with 4% formalin prepared in phosphate-buffered saline (PBS) and permeabilized with 0.5% Triton X-100 for 10 min. Cells were then rinsed and mounted with Everbrite Hardset Mounting Medium with DAPI (Biotium # 23004).

Receptor internalization assay (whole cell ELISA)
Changes in the levels of cell surface receptor (PTHR1-6E) were determined in at least three independent experiments, performed in technical duplicates, using whole cell ELISA. Cells expressing PTHR1-6E (or negative control cells) were seeded into poly-L-Lysine-coated 6-well plates and incubated overnight at 37°C. Treatment compounds were prepared in cell culture medium to achieve desired concentrations. Compounds were added to cells, which were incubated for 30 min at 37°C. After incubation and washing, receptor trafficking was quenched by placing cells on ice and performing fixation with 4% paraformaldehyde in PBS for 15 min. Fixed cells were washed twice with PBS and blocked with 2% BSA in PBS for 1 h at RT. Cell surface PTHR1-6E was detected using Nb_{6E}-biotin (30 nM) diluted in blocking solution. Nb_{6E}-biotin was incubated with fixed cells for 30 min. Following washing, horseradish peroxidase-conjugated streptavidin (Pierce #21130, 1:2000 dilution) was applied to the cells and incubated for 30 min at RT. After washing, the cells were exposed to one-step TMB-ELISA solution (Thermo Scientific #34028) and incubated until visible color developed. 100 µL of developed solution was transferred to a 96-well plate, and analyzed by recording absorbance at 405 nm using a microplate reader.

Surface Plasmon Resonance
SPR measurements were performed on a GE Biacore T200 Sensitivity Enhanced Instrument using a Cytiva Series S Sensor Chip SA (immobilized streptavidin). Ligand (Nb_{PTHR1}-biotin or Nb_{GLP1R}-biotin) was prepared using standard sortase ligation protocols described in the methods and diluted to a concentration of 0.1 µg/mL. These solutions were to immobilize Nb-biotin conjugates onto sensor chips. Analyte samples (PTHR1- or GLP1R-ECD) were prepared via two-fold serial dilutions in PBST ranging from 2 µM to 0.6 nM. Analyte was flowed over the chip at 50 µL/min with a contact time 60 seconds, and a dissociation time of 300 sec. The regeneration step (to dissociate receptor ECD) involved consecutive washes with a 10 mM glycine solution (pH 1.5).

Sensorgrams were fitted using the Biacore T200 evaluation software to a 1:1 binding model, with local Rmax. Reported K_D values incorporate affinities derived from association and dissociation measurements performed with multiple concentrations of receptor ECDs.

Circular Dichroism (CD)

CD spectra of peptide and ligand conjugates (at a concentration of 100 μM in TBS) were recorded at 25 °C on a JASCO J-720 spectropolarimeter using a 0.05-cm path length quartz cell. Peptide, Nb, and conjugate concentrations were determined measuring the absorbance at 280 nm. Spectra were recorded over a 180–260 nm range .

cAMP signaling assay for SaOS2 cells

SaOS2 (human osteosarcoma) cells stably expressing the Glosensor cAMP reporter[7], were maintained in modified McCoy's 5A medium supplemented with 15% FBS, 1% penicillin/streptomycin, and 1% nonessential amino acids. To assess ligand-induced cAMP signaling responses, intact cells were seeded in a white 96-well plate and used after 3-5 days of reaching confluency. At the time of the assay, the cells were preloaded with luciferin (0.5 mM) containing CO_2 independent media and incubated for 30 minutes at room temperature. Subsequently, cells were loaded with varying concentration of ligands and luminescence (as counts per second, cps), was recorded at 2-minute intervals in a Perkin Elmer Envision plate reader for 30 minutes (ligand-on phase). For washout assays, the plates were removed from the machine and the cells were rinsed twice with media to remove free ligands, new media containing luciferin was added, and luminescence was recorded at 2-minute intervals for 90 minutes (washout phase). The maximal luminescence response (observed 12–20 min after ligand addition) was used for generating dose-response curves. For washout responses, area under curve was generated from the time-course luminescence responses measured at multiple doses.

<u>Supplementary References:</u>

(1) Braun, M.B., et al. "Peptides in headlock–a novel high-affinity and versatile peptide-binding nanobody for proteomics and microscopy." *Scientific reports* 6.1:19211 (2016).

(2) Rajagopal, S., et al. "Quantifying ligand bias at seven-transmembrane receptors." *Molecular pharmacology* 80.3: 367-377 (2011).

(3) Kubala, M. H.; Kovtun, O.; Alexandrov, K.; Collins, B. M. Structural and Thermodynamic Analysis of the GFP:GFP-Nanobody Complex. *Protein Sci. Publ. Protein Soc*, *19* (12), 2389–2401 (2010).

(4) Tabibiazar, R., Sato, A., Garg, P., Liu, Q. and Axelrod, F., Twist Bioscience Corp, *Variant nucleic acid libraries for GLP1 receptor*. U.S. Patent 11,492,727 (2022).

(5) Adelhorst, K., Hedegaard, B.B., Knudsen, L.B. and Kirk, O. Structure-activity studies of glucagon-like peptide-1. *Journal of Biological Chemistry*, *269*(9), pp.6275-6278 (1994).

(6) Sato, A., Garg, P., Liu, Q. and Axelrod, F., Twist Bioscience Corp., *Methods and compositions relating to glp1r variants*. U.S. Patent Application 17/412,139 (2022).

(7) Cullum, S.A., Veprintsev, D.B. and Hill, S.J. Kinetic analysis of endogenous β2-adrenoceptor-mediated cAMP GloSensor™ responses in HEK293 cells. *British Journal of Pharmacology*, *180*(10), pp.1304-1315 (2023).

(8) Cheloha, RW., et al. "Improved GPCR ligands from nanobody tethering." *Nature Communications* 11.1: 2087 (2020).

(9) Fan, F., et al. "Novel genetically encoded biosensors using firefly luciferase." *ACS chemical biology* 3.6: 346-351 (2008).
