# Supplementary material for: Highly biased agonism for GPCR ligands via nanobody tethering
Source: Nat Commun. 2024 Jun 1;15:4687. doi: 10.1038/s41467-024-49068-5 (PMC11144202; doi:10.1038/s41467-024-49068-5)
Supplement: Supplementary file 3 — Description of Additional Supplementary Files [file 41467_2024_49068_MOESM3_ESM.pdf]

File name: Supplementary Data 1
Description: Mass spectrometry characterization of Nb-peptide conjugates used in this study. Compounds were analyzed by liquid chromatography/mass spectrometry as described in Methods.
