# Supplementary material for: Highly biased agonism for GPCR ligands via nanobody tethering
Source: Nat Commun. 2024 Jun 1;15:4687. doi: 10.1038/s41467-024-49068-5 (PMC11144202; doi:10.1038/s41467-024-49068-5)
Supplement: Supplementary file 4 — Supplementary Data 1 [file 41467_2024_49068_MOESM4_ESM.pdf]

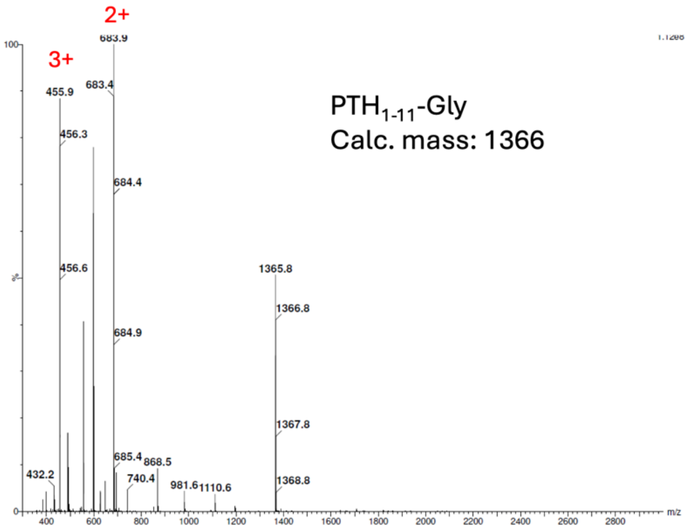
PTH1-11-Gly
Calc. mass: 1366
2+
683.9
3+
455.9
683.4
456.3
684.4
456.6
1365.8
1366.8
684.9
1367.8
685.4
868.5
432.2
740.4
981.6
1110.6
1368.8
m/z

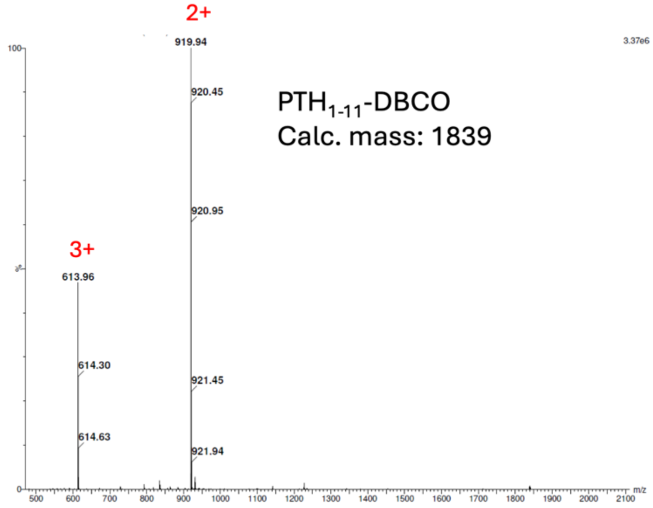
PTH1-11-DBCO
Calc. mass: 1839
2+
919.94
920.45
920.95
3+
613.96
614.30
614.63
921.45
921.94
3.37e6
m/z

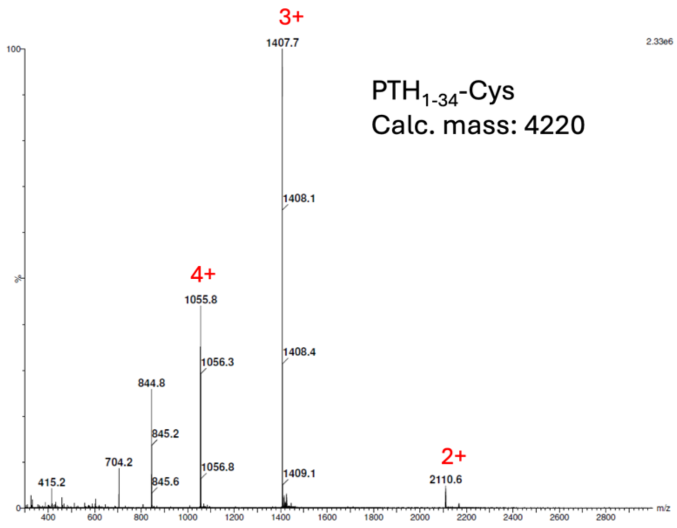
PTH1-34-Cys
Calc. mass: 4220
3+
1407.7
1408.1
4+
1055.8
1056.3
844.8
1408.4
845.2
704.2
1056.8
415.2
845.6
1409.1
2+
2110.6
2.33e6
m/z

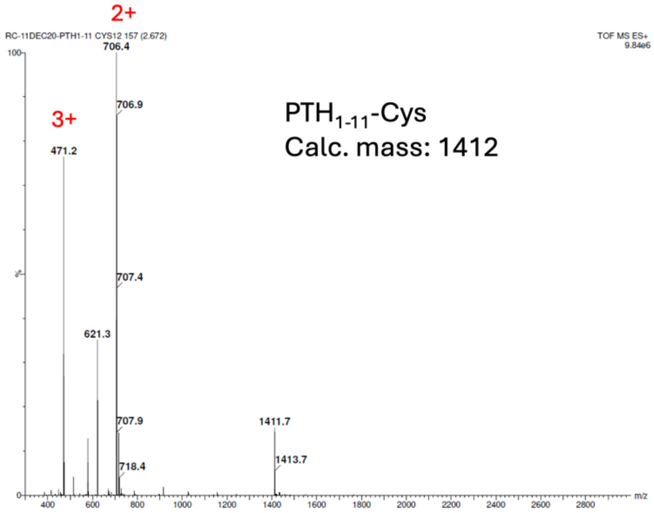
PTH1-11-Cys
Calc. mass: 1412
2+
706.4
706.9
3+
471.2
707.4
621.3
707.9
1411.7
1413.7
718.4
TOF MS ES+
9.84e6
m/z

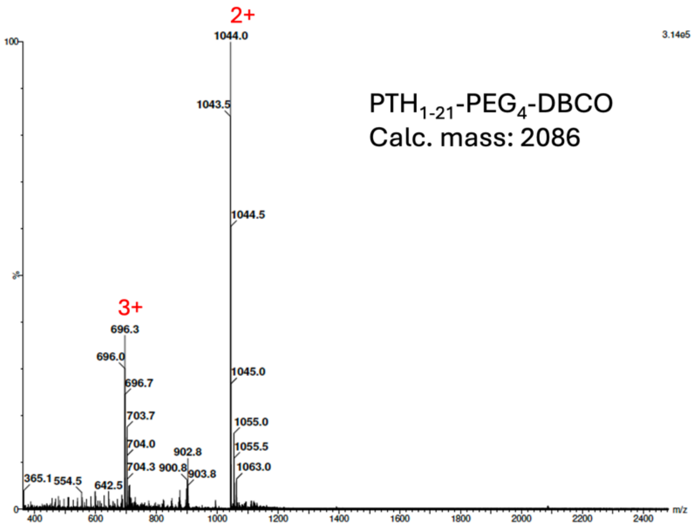
PTH1-21-PEG4-DBCO
Calc. mass: 2086
2+
1044.0
1043.5
1044.5
3+
696.3
696.0
696.7
1045.0
703.7
1055.0
704.0
1055.5
902.8
704.3
900.8
903.8
1063.0
365.1
554.5
642.5
3.14e5
m/z

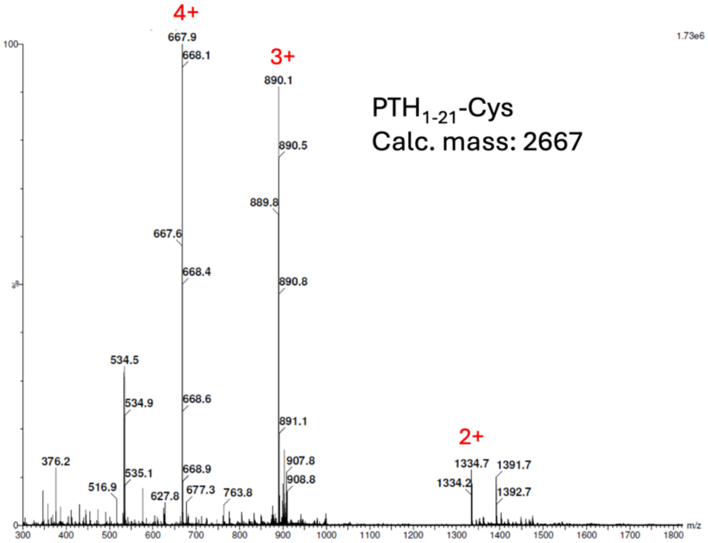
PTH1-21-Cys
Calc. mass: 2667
4+
667.9
668.1
3+
890.1
890.5
889.8
667.6
668.4
890.8
534.5
534.9
668.6
891.1
2+
376.2
907.8
1334.7
1391.7
908.8
516.9
535.1
668.9
1334.2
1392.7
627.8
677.3
763.8
1.73e6
m/z

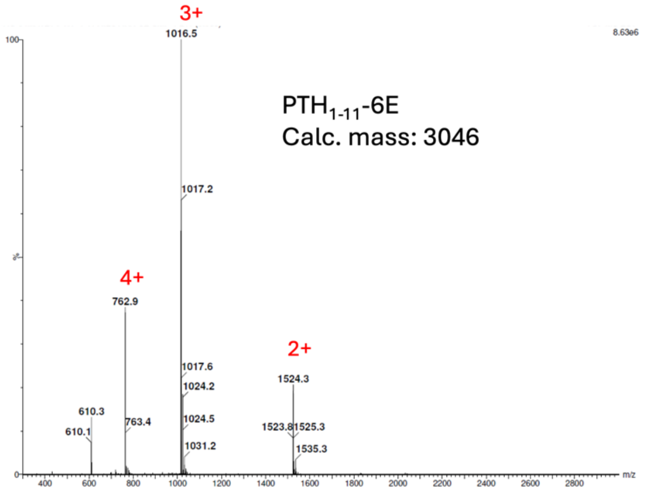
PTH1-11-6E
Calc. mass: 3046
3+
1016.5
1017.2
1017.6
1024.2
1024.5
1031.2
4+
762.9
763.4
610.3
610.1
2+
1524.3
1523.8
1525.3
1535.3
8.63e6
m/z

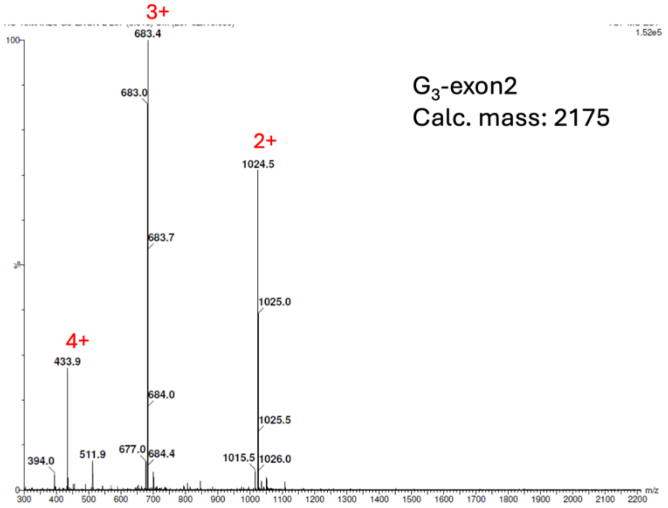
G3-exon2
Calc. mass: 2175
3+
683.4
683.0
683.7
684.0
684.4
677.0
2+
1024.5
1025.0
1025.5
1026.0
1015.5
4+
433.9
394.0
511.9
1.52e5
m/z

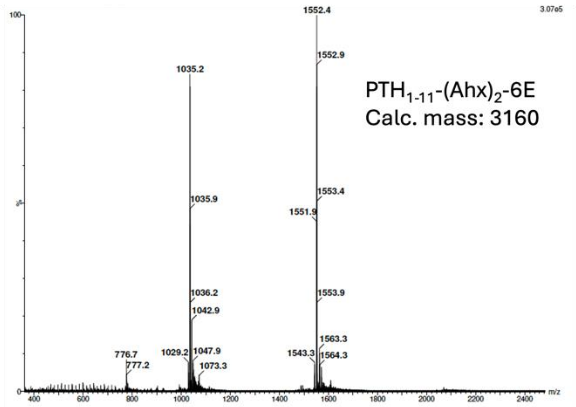
PTH1-11-(Ahx)2-6E
Calc. mass: 3160
1552.4
1552.9
1553.4
1551.9
1553.9
1563.3
1564.3
1543.3
1035.2
1035.9
1036.2
1042.9
1029.2
1047.9
1073.3
776.7
777.2
3.07e5
m/z

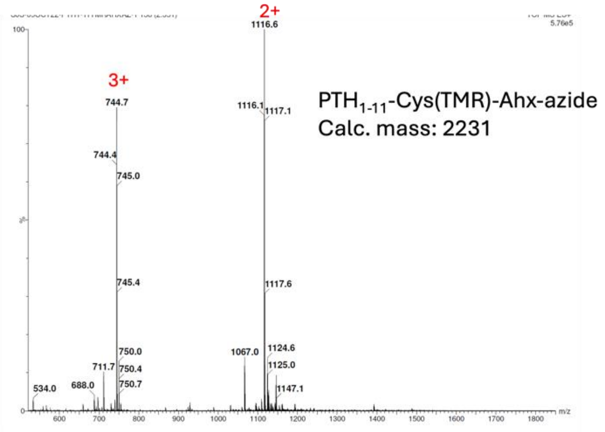
PTH1-11-Cys(TMR)-Ahx-azide
Calc. mass: 2231
2+
1116.6
1116.1
1117.1
1117.6
1124.6
1125.0
1147.1
1067.0
3+
744.7
744.4
745.0
745.4
750.0
750.4
750.7
711.7
688.0
534.0
5.76e5
m/z

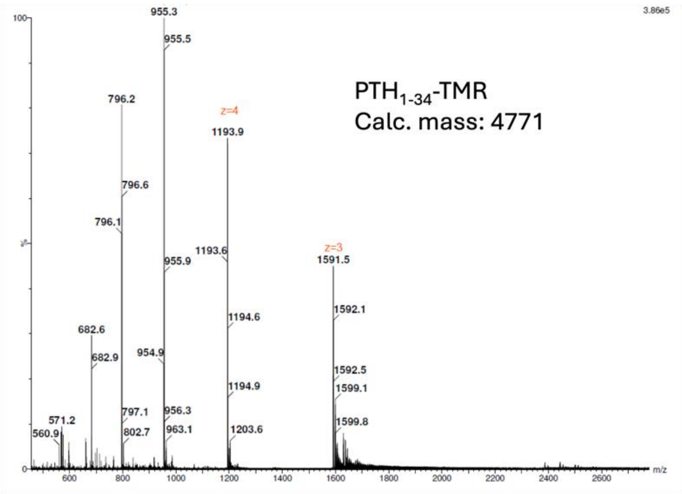
PTH1-34-TMR
Calc. mass: 4771
955.3
955.5
955.9
954.9
956.3
963.1
796.2
796.6
796.1
797.1
802.7
z=4
1193.9
1193.6
1194.6
1194.9
1203.6
z=3
1591.5
1592.1
1592.5
1599.1
1599.8
682.6
682.9
571.2
560.9
3.86e5
m/z

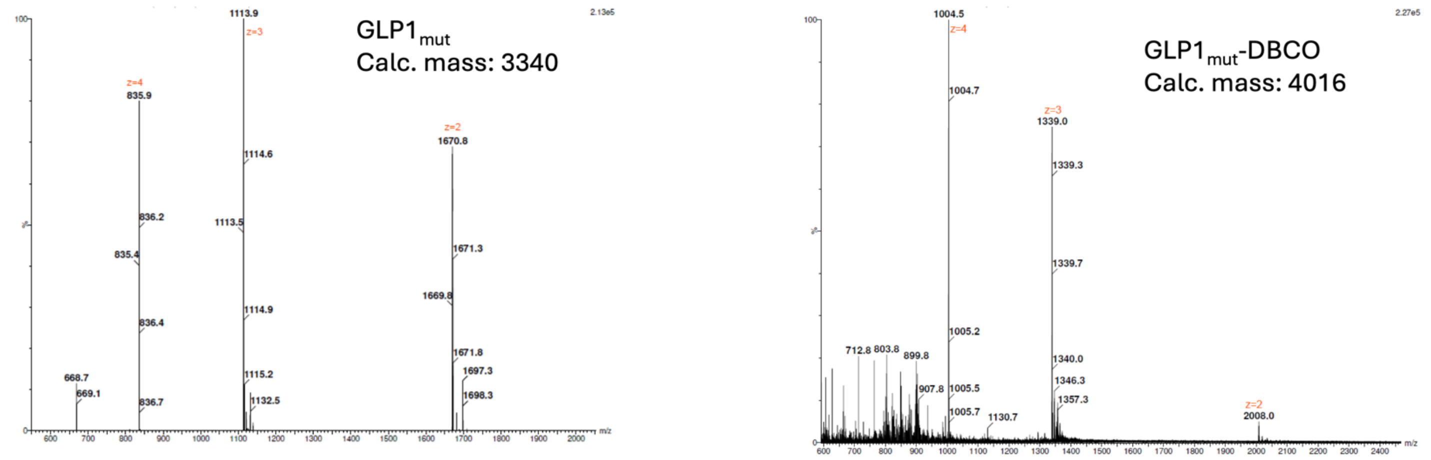

Mass spectrometry characterization of peptides used in this study. Compounds were analyzed by liquid chromatography/mass spectrometry as described in Methods.

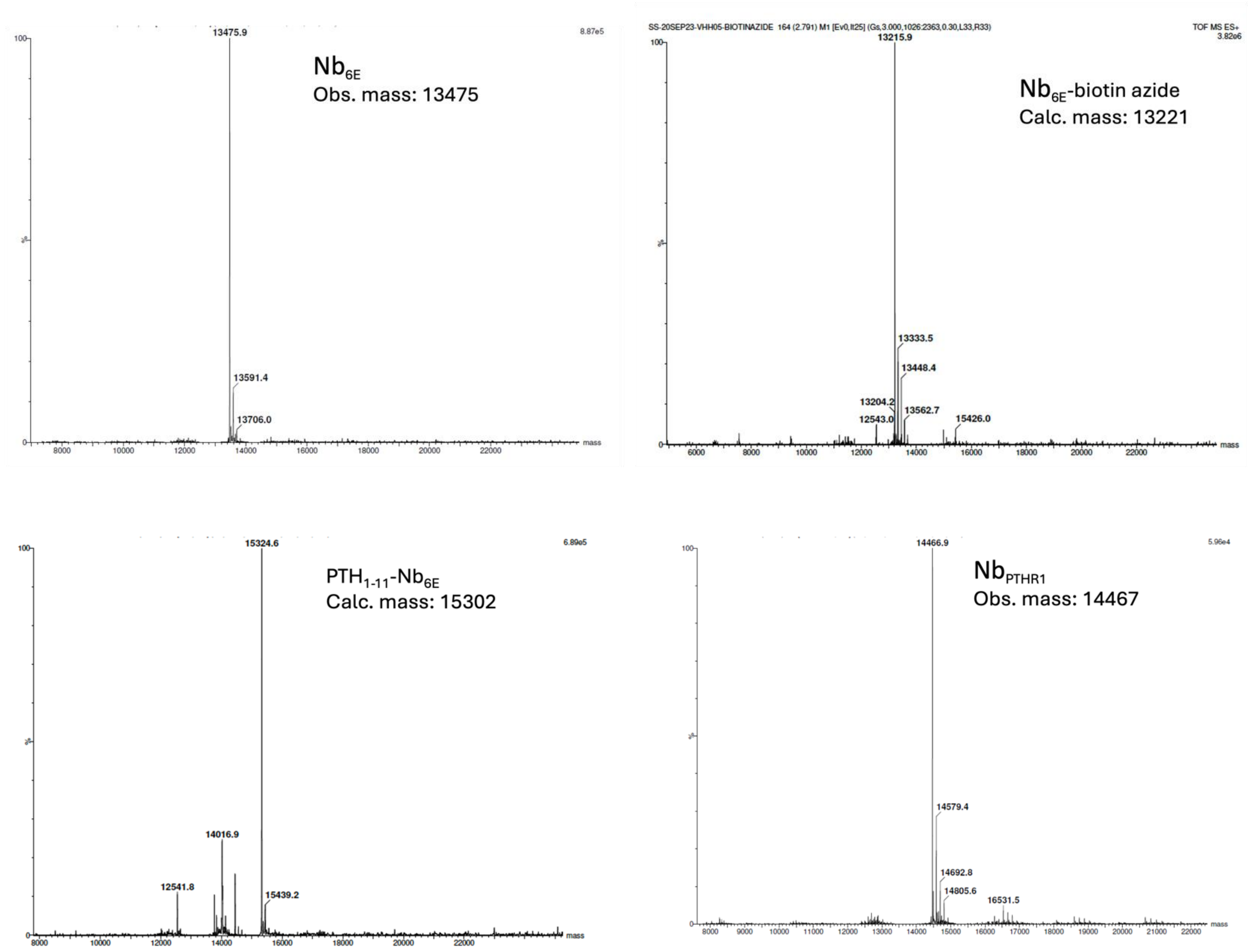

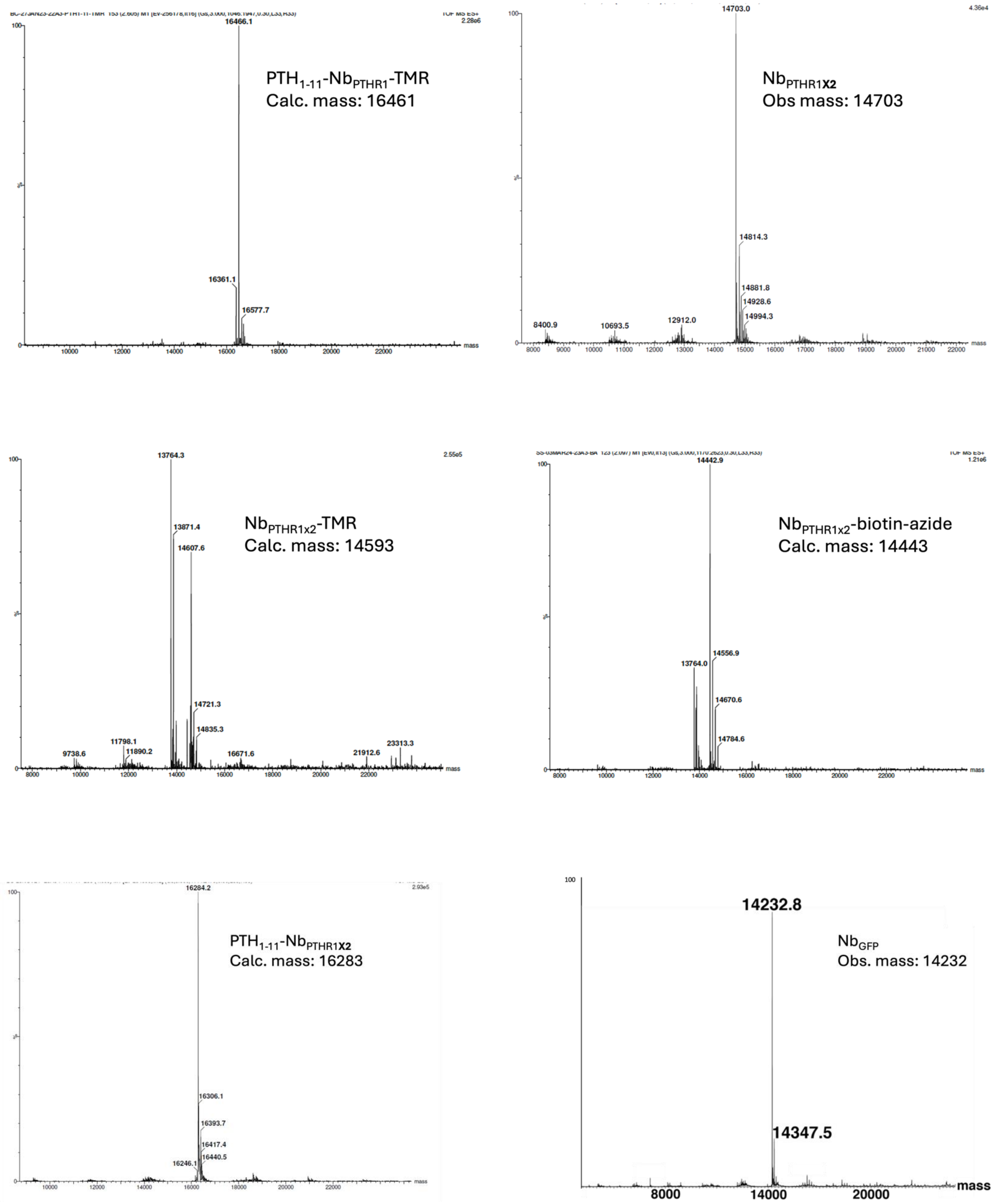
PTH1-11-NbPTHR1-TMR
Calc. mass: 16461
16466.1
16361.1
16577.7
mass
NbPTHR1X2
Obs mass: 14703
14703.0
14814.3
14881.8
14928.6
14994.3
8400.9
10693.5
12912.0
mass
NbPTHR1x2-TMR
Calc. mass: 14593
13764.3
13871.4
14607.6
14721.3
14835.3
9738.6
11798.1
11890.2
16671.6
21912.6
23313.3
mass
NbPTHR1x2-biotin-azide
Calc. mass: 14443
14442.9
13764.0
14556.9
14670.6
14784.6
mass
PTH1-11-NbPTHR1X2
Calc. mass: 16283
16284.2
16306.1
16393.7
16417.4
16440.5
16246.1
mass
NbGFP
Obs. mass: 14232
14232.8
14347.5
mass

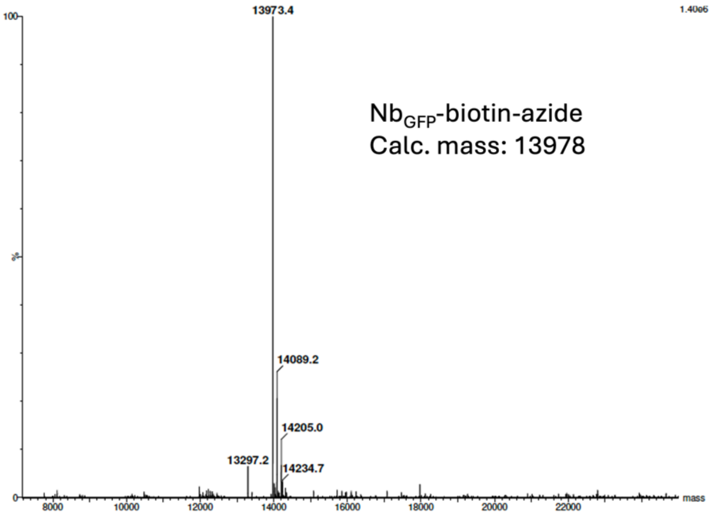
Nb$_{GFP}$-biotin-azide
Calc. mass: 13978
13973.4
14089.2
14205.0
14234.7
13297.2
1.40e6
100
%
0
8000
10000
12000
14000
16000
18000
20000
22000
mass

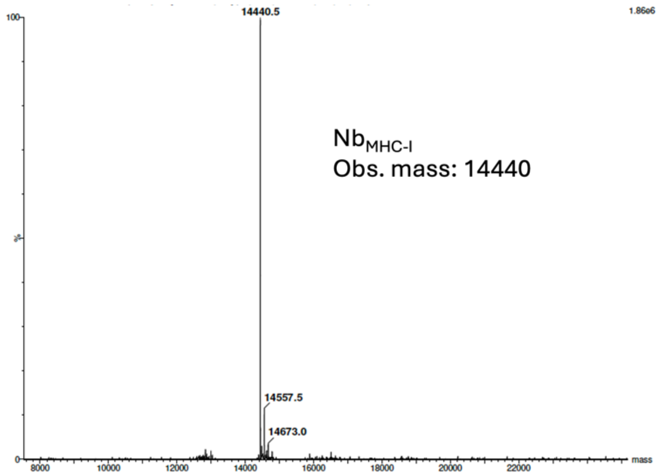
Nb$_{MHC-I}$
Obs. mass: 14440
14440.5
14557.5
14673.0
1.86e6
100
%
0
8000
10000
12000
14000
16000
18000
20000
22000
mass

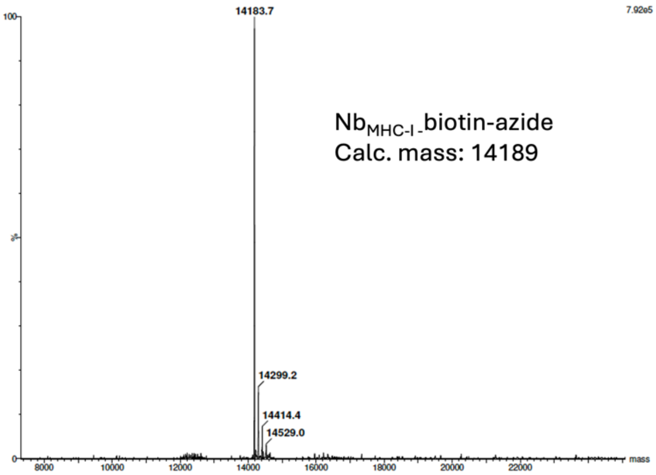
Nb$_{MHC-I}$-biotin-azide
Calc. mass: 14189
14183.7
14299.2
14414.4
14529.0
7.92e5
100
%
0
8000
10000
12000
14000
16000
18000
20000
22000
mass

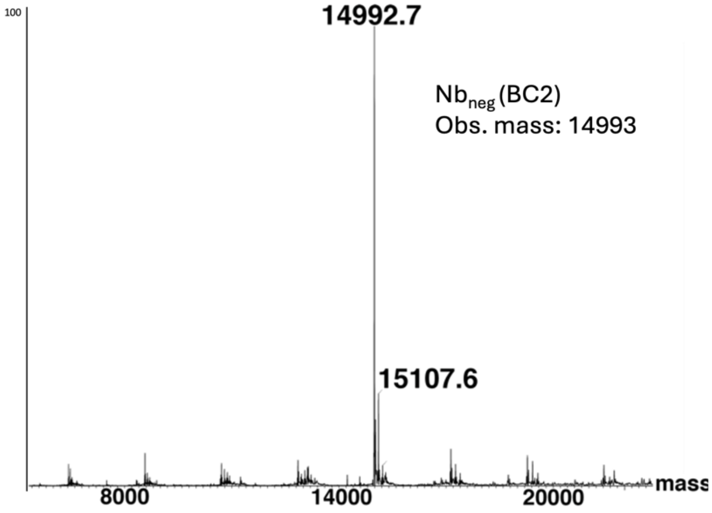
Nb$_{neg}$(BC2)
Obs. mass: 14993
14992.7
15107.6
100
8000
14000
20000
mass

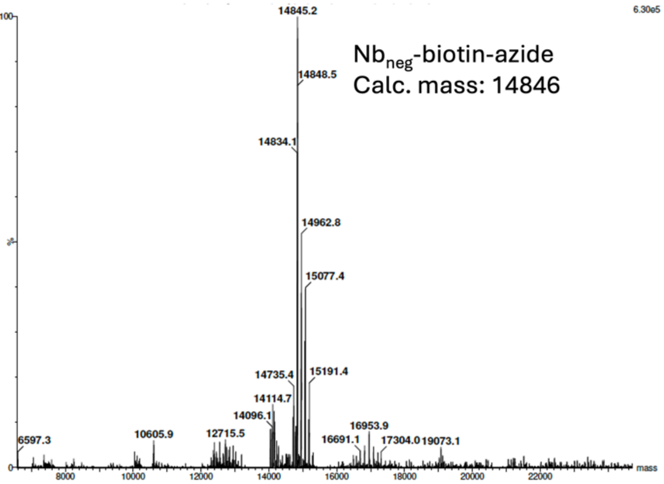
Nb$_{neg}$-biotin-azide
Calc. mass: 14846
14845.2
14848.5
14834.1
14962.8
15077.4
14735.4
15191.4
14114.7
14096.1
16953.9
6597.3
10605.9
12715.5
16691.1
17304.0
19073.1
6.30e5
100
%
0
8000
10000
12000
14000
16000
18000
20000
22000
mass

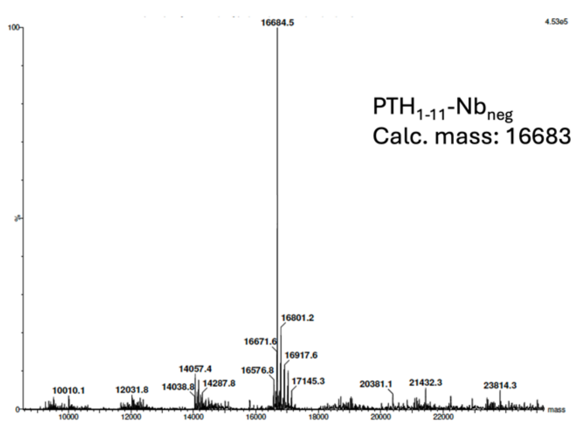

PTH_{1-11}-Nb_{neg}
Calc. mass: 16683
16684.5
4.53e5
16801.2
16671.6
16917.6
16576.8
17145.3
14057.4
14038.8
14287.8
10010.1
12031.8
20381.1
21432.3
23814.3
mass

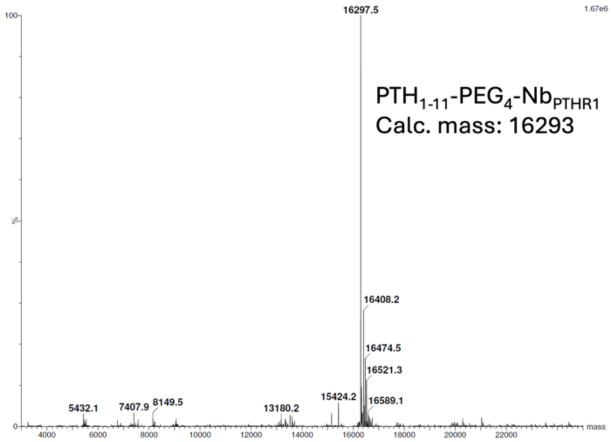

PTH_{1-11}-PEG_4-Nb_{PTHR1}
Calc. mass: 16293
16297.5
1.67e6
16408.2
16474.5
16521.3
16589.1
15424.2
13180.2
5432.1
7407.9
8149.5
mass

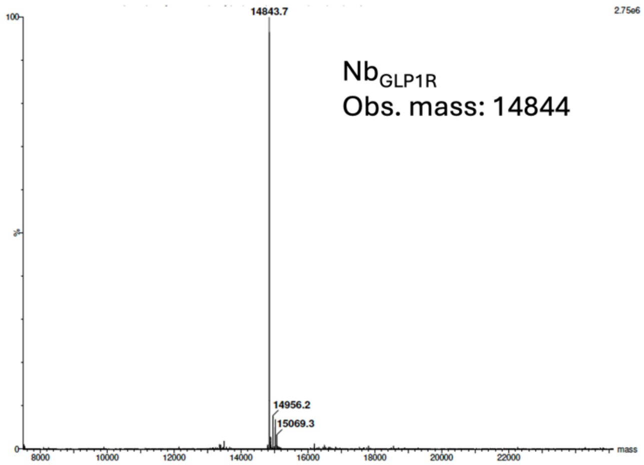

Nb_{GLP1R}
Obs. mass: 14844
14843.7
2.75e6
14956.2
15069.3
mass

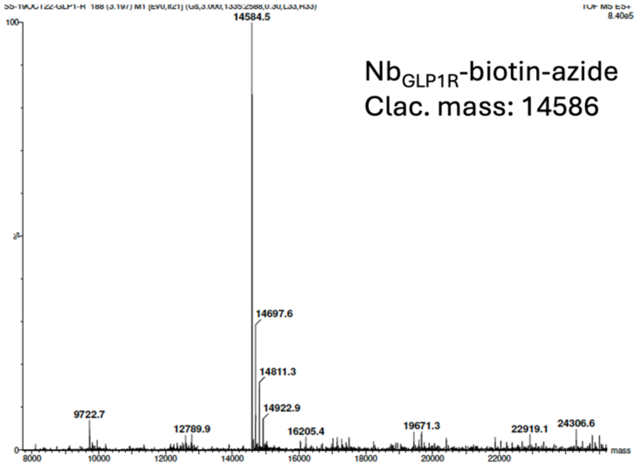

Nb_{GLP1R}-biotin-azide
Clac. mass: 14586
14584.5
8.40e5
14697.6
14811.3
14922.9
9722.7
12789.9
16205.4
19671.3
22919.1
24306.6
mass

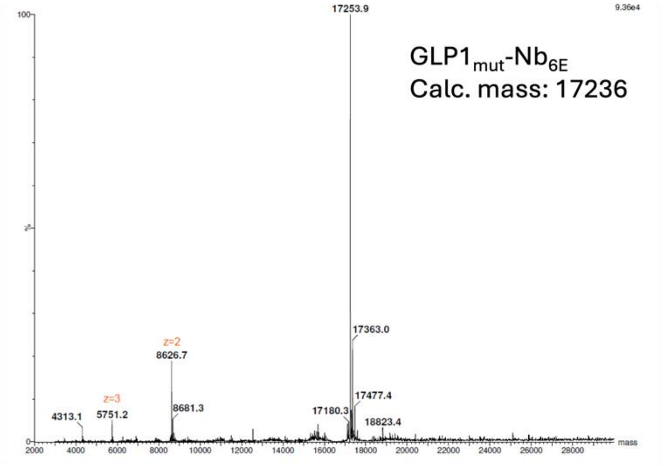

$GLP1_{mut}$-Nb_{6E}
Calc. mass: 17236
17253.9
9.36e4
17363.0
17477.4
17180.3
18823.4
z=2
8626.7
8681.3
z=3
5751.2
4313.1
mass

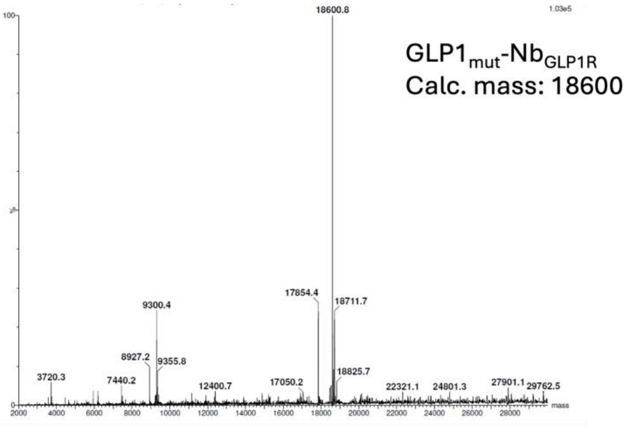

$GLP1_{mut}$-Nb_{GLP1R}
Calc. mass: 18600
18600.8
1.03e5
17854.4
18711.7
18825.7
17050.2
9300.4
8927.2
9355.8
7440.2
3720.3
12400.7
22321.1
24801.3
27901.1
29762.5
mass

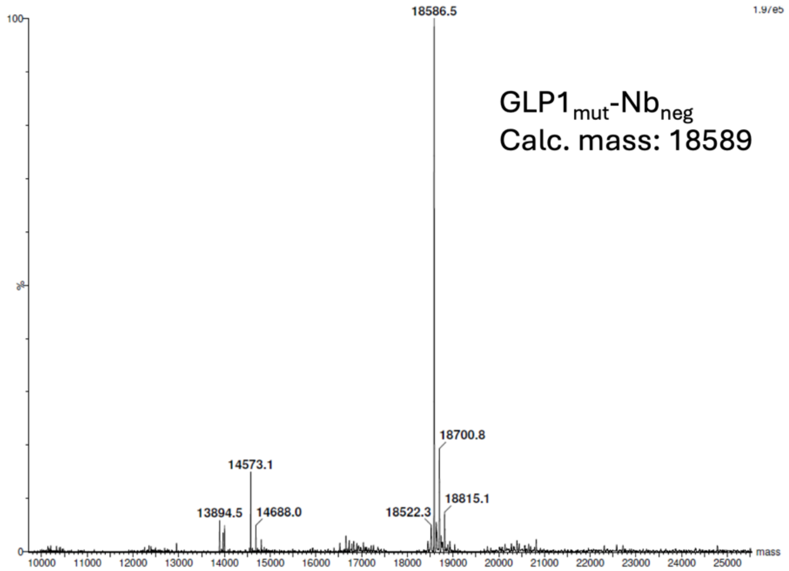

Mass spectrometry characterization of Nb-peptide conjugates used in this study. Compounds were analyzed by liquid chromatography/mass spectrometry as described in Methods.
