# Supplementary material for: Highly biased agonism for GPCR ligands via nanobody tethering
Source: Nat Commun. 2024 Jun 1;15:4687. doi: 10.1038/s41467-024-49068-5 (PMC11144202; doi:10.1038/s41467-024-49068-5)
Supplement: Supplementary file 5 — Reporting Summary [file 41467_2024_49068_MOESM5_ESM.pdf]

nature portfolio

Corresponding author(s): Ross Cheloha

Last updated by author(s): Mar 11, 2024

Reporting Summary

Nature Portfolio wishes to improve the reproducibility of the work that we publish. This form provides structure for consistency and transparency in reporting. For further information on Nature Portfolio policies, see our Editorial Policies and the Editorial Policy Checklist.

Statistics

For all statistical analyses, confirm that the following items are present in the figure legend, table legend, main text, or Methods section.

n/a	Confirmed	
☐	☒	The exact sample size (*n*) for each experimental group/condition, given as a discrete number and unit of measurement
☐	☒	A statement on whether measurements were taken from distinct samples or whether the same sample was measured repeatedly
☐	☒	The statistical test(s) used AND whether they are one- or two-sided<br>*Only common tests should be described solely by name; describe more complex techniques in the Methods section.*
☒	☐	A description of all covariates tested
☐	☒	A description of any assumptions or corrections, such as tests of normality and adjustment for multiple comparisons
☐	☒	A full description of the statistical parameters including central tendency (e.g. means) or other basic estimates (e.g. regression coefficient) AND variation (e.g. standard deviation) or associated estimates of uncertainty (e.g. confidence intervals)
☐	☒	For null hypothesis testing, the test statistic (e.g. *F*, *t*, *r*) with confidence intervals, effect sizes, degrees of freedom and *P* value noted<br>*Give P values as exact values whenever suitable.*
☒	☐	For Bayesian analysis, information on the choice of priors and Markov chain Monte Carlo settings
☒	☐	For hierarchical and complex designs, identification of the appropriate level for tests and full reporting of outcomes
☒	☐	Estimates of effect sizes (e.g. Cohen's *d*, Pearson's *r*), indicating how they were calculated

Our web collection on statistics for biologists contains articles on many of the points above.

Software and code

Policy information about availability of computer code

Data collection	N/A
Data analysis	Graphpad Prism (v10), Microsoft Excel, Pymol, and FlowJo (v7) were used for data analysis and visualization

For manuscripts utilizing custom algorithms or software that are central to the research but not yet described in published literature, software must be made available to editors and reviewers. We strongly encourage code deposition in a community repository (e.g. GitHub). See the Nature Portfolio guidelines for submitting code & software for further information.

Data

Policy information about availability of data

All manuscripts must include a data availability statement. This statement should provide the following information, where applicable:

- Accession codes, unique identifiers, or web links for publicly available datasets
- A description of any restrictions on data availability
- For clinical datasets or third party data, please ensure that the statement adheres to our policy

All data is available in the manuscript and supporting information

Research involving human participants, their data, or biological material

Policy information about studies with human participants or human data. See also policy information about sex, gender (identity/presentation), and sexual orientation and race, ethnicity and racism.

Reporting on sex and gender	N/A
Reporting on race, ethnicity, or other socially relevant groupings	N/A
Population characteristics	N/A
Recruitment	N/A
Ethics oversight	N/A

Note that full information on the approval of the study protocol must also be provided in the manuscript.

Field-specific reporting

Please select the one below that is the best fit for your research. If you are not sure, read the appropriate sections before making your selection.

☒ Life sciences ☐ Behavioural & social sciences ☐ Ecological, evolutionary & environmental sciences

For a reference copy of the document with all sections, see nature.com/documents/nr-reporting-summary-flat.pdf

Life sciences study design

All studies must disclose on these points even when the disclosure is negative.

Sample size	No predetermination of sample size was performed
Data exclusions	Assays includes a compound (PTH1-34) that serves an internal control. Any failure of PTH1-34 to provide suitable results indicates a failed assay that is discarded in its entirety
Replication	All cell-based assays were reproduced 2-6 times, with the exact numbers of replicates listed in a Supporting Information table
Randomization	No randomization was performed
Blinding	No blinding was performed

Reporting for specific materials, systems and methods

We require information from authors about some types of materials, experimental systems and methods used in many studies. Here, indicate whether each material, system or method listed is relevant to your study. If you are not sure if a list item applies to your research, read the appropriate section before selecting a response.

Materials & experimental systems

n/a	Involved in the study	
☐	☒	Antibodies
☐	☒	Eukaryotic cell lines
☒	☐	Palaeontology and archaeology
☒	☐	Animals and other organisms
☒	☐	Clinical data
☒	☐	Dual use research of concern
☒	☐	Plants

Methods

n/a	Involved in the study	
☒	☐	ChIP-seq
☐	☒	Flow cytometry
☒	☐	MRI-based neuroimaging

Antibodies

Antibodies used	Single domain antibodies (nanobodies) produced in house are used in the manuscript, as described in methods

Validation	These nanobodies were validated in house by testing on cell lines expressing (or lacking) targets of interest with analysis by flow cytometry

Eukaryotic cell lines

Policy information about cell lines and Sex and Gender in Research

Cell line source(s)	HEK293-based cell lines. Originally obtained from ATCC and transfected to express target proteins of interest. Different clonal cell lines maintained by the Gardella lab (Massachusetts General Hospital) or Cheloha lab (National Insitutes of Health)<br>SaOS2 cell line--obtained from ATCC
Authentication	Cell lines were authenticated though analysis with flow cytometry
Mycoplasma contamination	Mycoplasma tests are conducted on a regular basis (2 times/year). No mycoplasma contamination was detected
Commonly misidentified lines<br>(See ICLAC register)	None

Plants

Seed stocks	N/A
Novel plant genotypes	N/A
Authentication	N/A

Flow Cytometry

Plots

Confirm that:

- [x] The axis labels state the marker and fluorochrome used (e.g. CD4-FITC).
- [x] The axis scales are clearly visible. Include numbers along axes only for bottom left plot of group (a 'group' is an analysis of identical markers).
- [ ] All plots are contour plots with outliers or pseudocolor plots.
- [ ] A numerical value for number of cells or percentage (with statistics) is provided.

Methodology

Sample preparation	Cell lines (HEK293) were trypsinized from culture plates, washed, and resuspended in PBS (BSA) before treating with antibodies/nanobodies with biotin or fluorophore attached. After staining for 1 h, cells were washed and then analyzed by flow cytometry
Instrument	CytoFlex flow cytometer (Beckman Coulter)
Software	FlowJo v7
Cell population abundance	Intact, live cells were selected and comprised between 10-90% of total events
Gating strategy	Forward scatter and side scatter was used to identify live cells for further analysis

- [x] Tick this box to confirm that a figure exemplifying the gating strategy is provided in the Supplementary Information.
